# Supplementary material for: Self-Assembly of Supramolecular Architectures by the Effect of Amino Acid Residues of Quaternary Ammonium Pillar[5]arenes
Source: Int J Mol Sci. 2020 Sep 29;21(19):7206. doi: 10.3390/ijms21197206 (PMC7582551; doi:10.3390/ijms21197206)

[1] Self-assembly of supramolecular architectures by the effect of amino acid residues of quaternary ammonium pillar[5]arenes

Anastasia Nazarova [1], Dmitriy Shurpik [1], Pavel Padnya [1], Timur Mukhametzyanov [1], Peter Cragg [2] and Ivan Stoikov [1,*]

[2] A.M.Butlerov Chemical Institute, Kazan Federal University, 420008 Kremlevskaya, 18, Kazan, Russian Federation; anas7tasia@gmail.com (A.N.); dnshurpik@mail.ru (D.S.); padnya.ksu@gmail.com (P.P.); timmie.m@gmail.com (T.M.).

[3] School of Pharmacy and Biomolecular Sciences, University of Brighton, Huxley Building, Moulsecoomb. Brighton, East Sussex BN2 4GJ, UK; p.j.cragg@brighton.ac.uk (P. C.).

* Correspondence: ivan.stoikov@mail.ru; Tel.: +7-843-233-7241

Electronic Supplementary Information (49 pages)

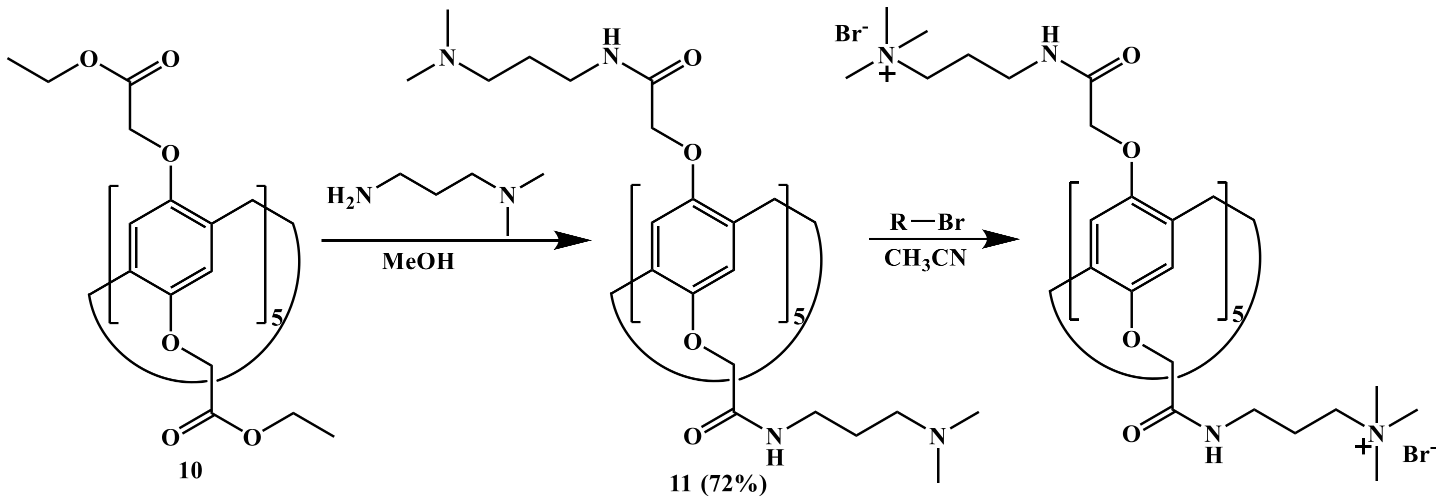

12: R = CH_2CO-Gly-OEt (89%);
13: R = CH_2CO-Ala-OEt (93%);
14: R = CH_2CO-GlyGly-OEt (90%);
15: R = CH_2CO-Phe-OEt (87%)

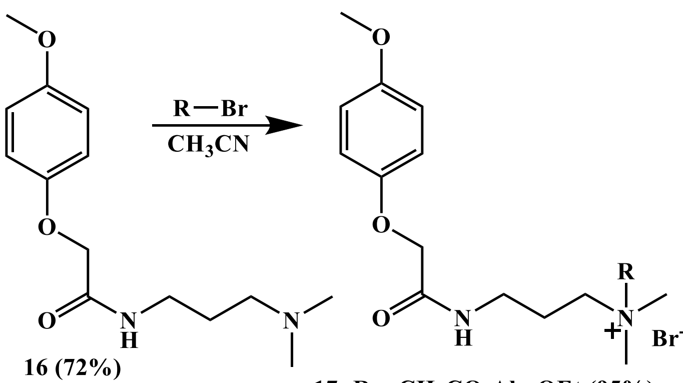

17: R = CH_2CO-Ala-OEt (95%);
18: R = CH_2CO-Phe-OEt (94%)

Fig. S1. ^{1}H NMR spectrum of 4,8,14,18,23,26,28,31,32,35-deca[(ethoxycarbonyl)methoxy]-pillar[5]arene *(10)*, $CDCl_3$, 298 K, 400 MHz.

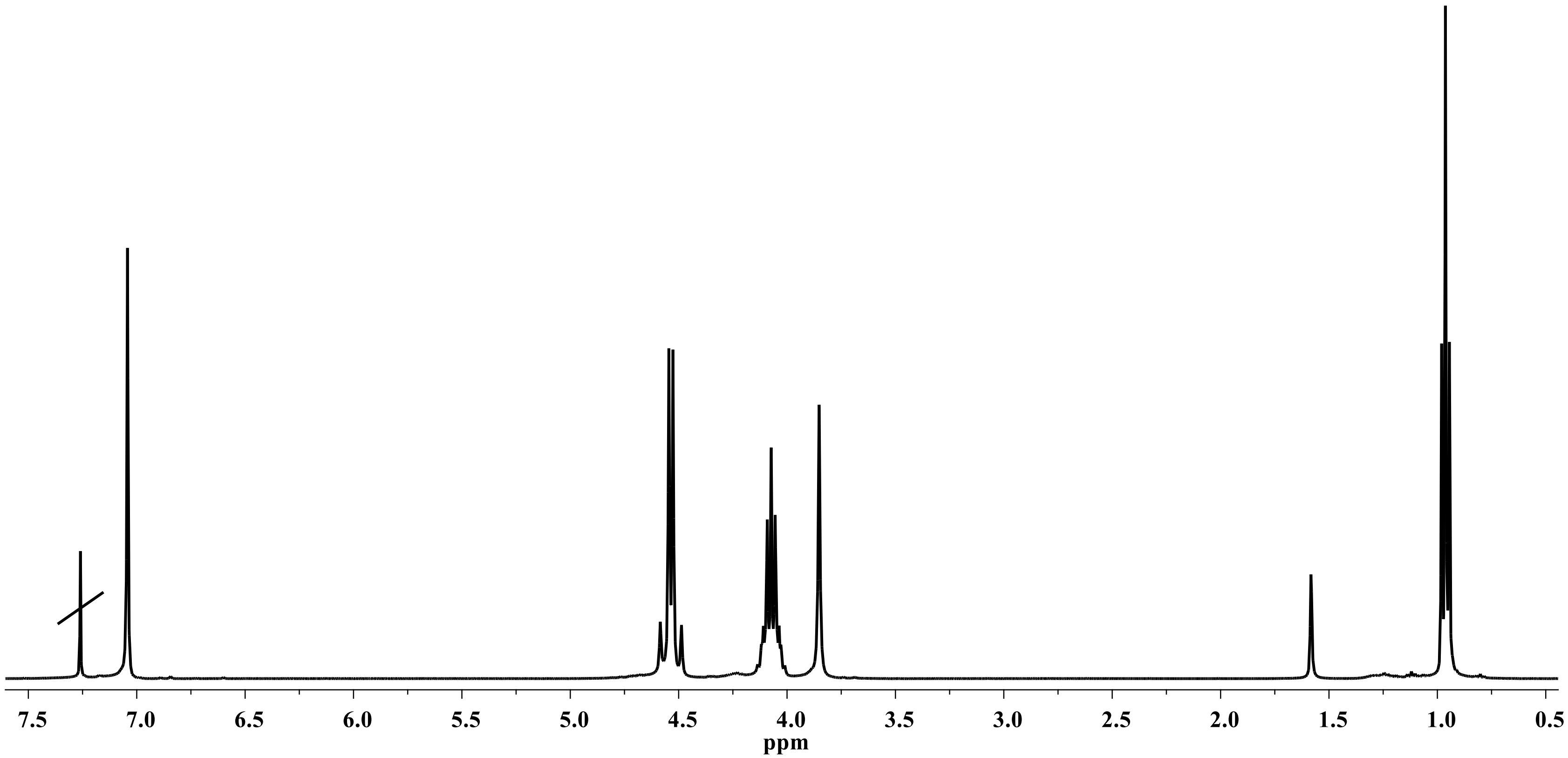

Fig. S2. [1]**H NMR spectrum of 4,8,14,18,23,26,28,31,32,35-deca-[(*N*-3′,3′-dimethylaminopropyl)-carbomoylmethoxy]-pillar[5]arene *(11)*, DMSO-*d*$_6$, 298 K, 400 MHz.**

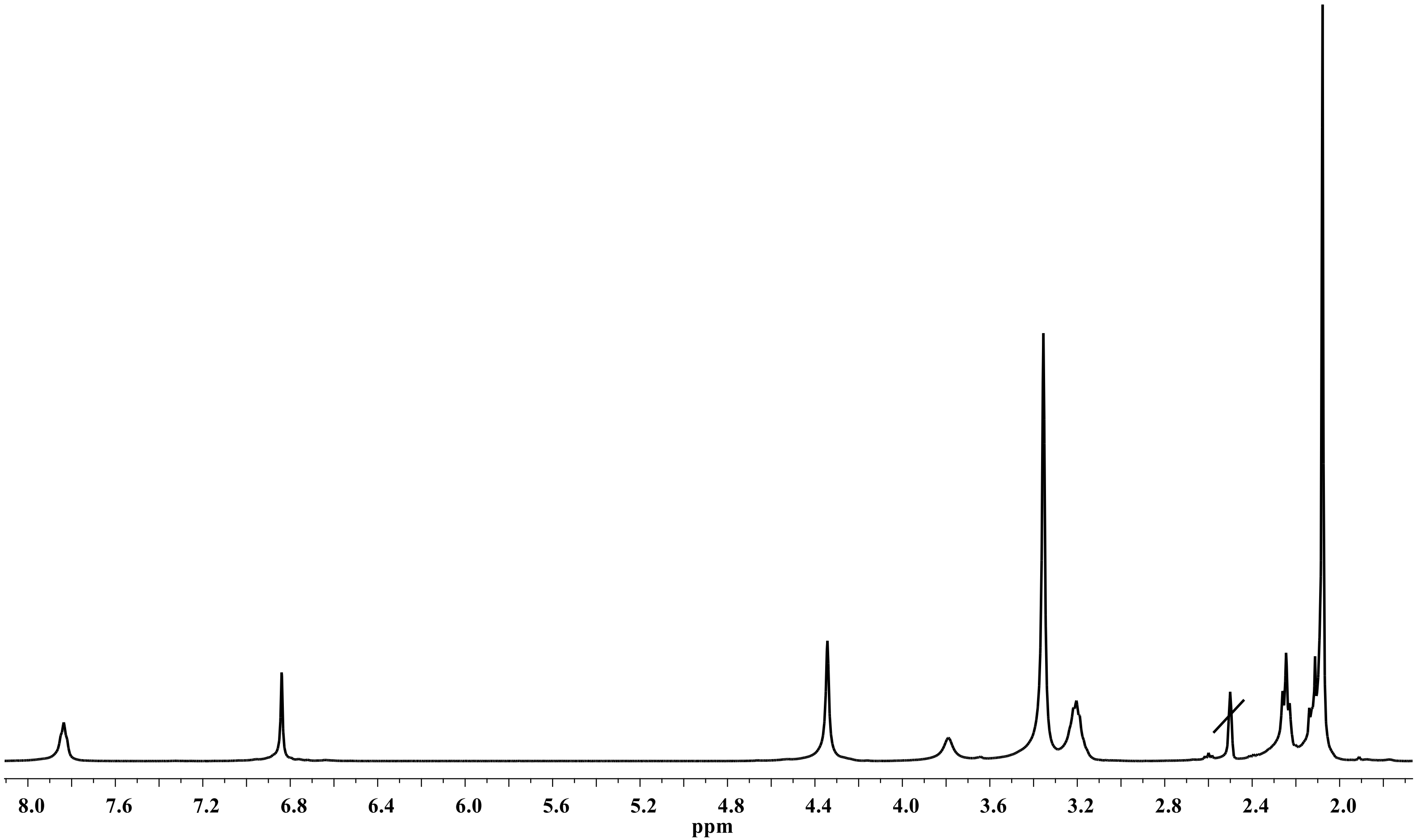

Fig. S3. ^{1}**H NMR spectrum of 4,8,14,18,23,26,28,31,32,35-deca-[(*N*-(3′,3′-dimethyl-3′-{(ethoxycarbonylmethyl)amidocarbonylmethyl}ammoniumpropyl)carbomoyl-methoxy]-pillar[5]arene decabromide *(12)*, DMSO-*d*$_6$, 298 K, 400 MHz.**

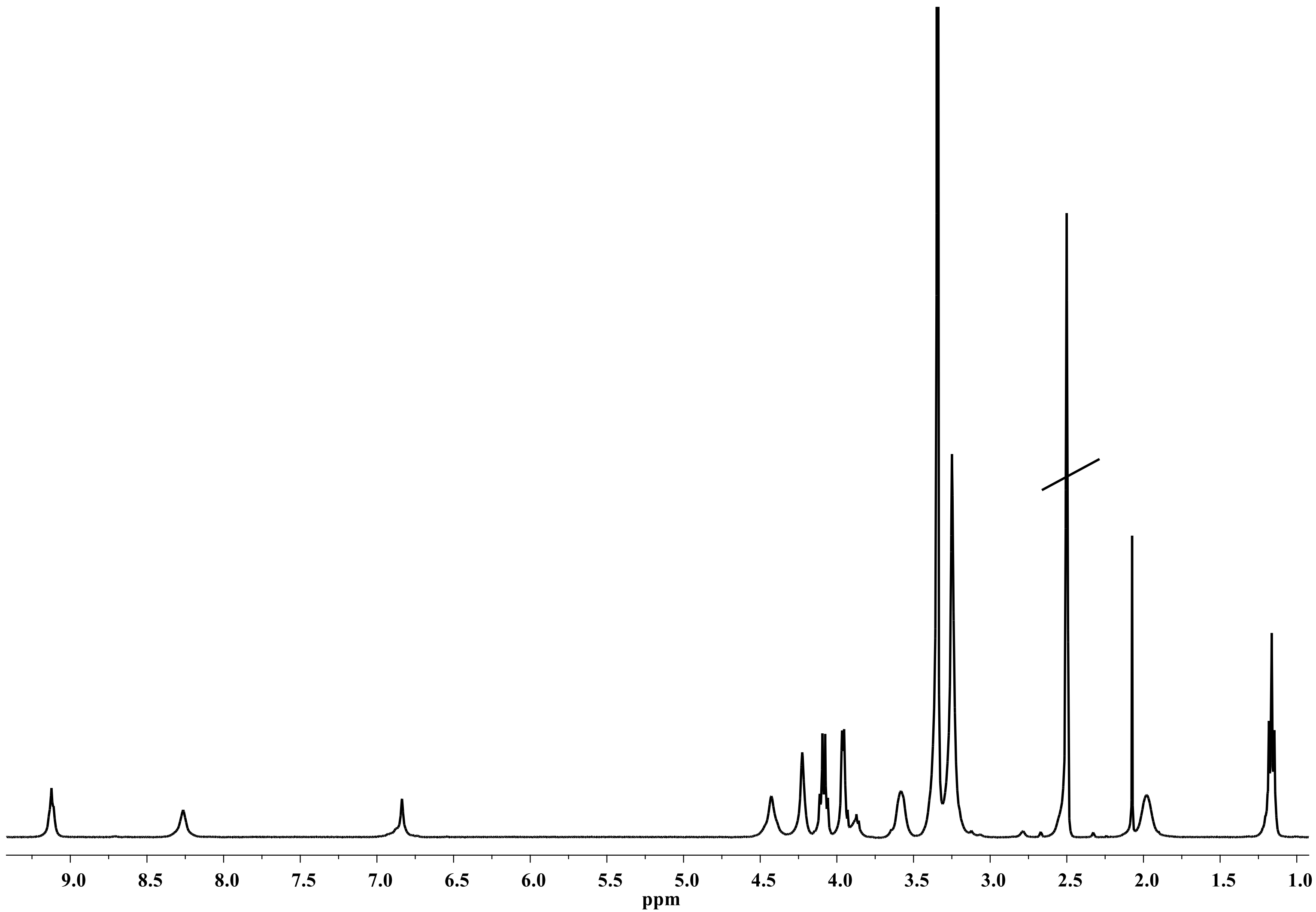

Fig. S4. ^1H NMR spectrum of 4,8,14,18,23,26,28,31,32,35-deca-[(*N*-(3′,3′-dimethyl-3′-{(ethoxycarbonylmethyl)amidocarbonylmethyl}ammoniumpropyl)carbomoyl-methoxy]-pillar[5]arene decabromide *(12)*, D_2O, 298 K, 400 MHz.

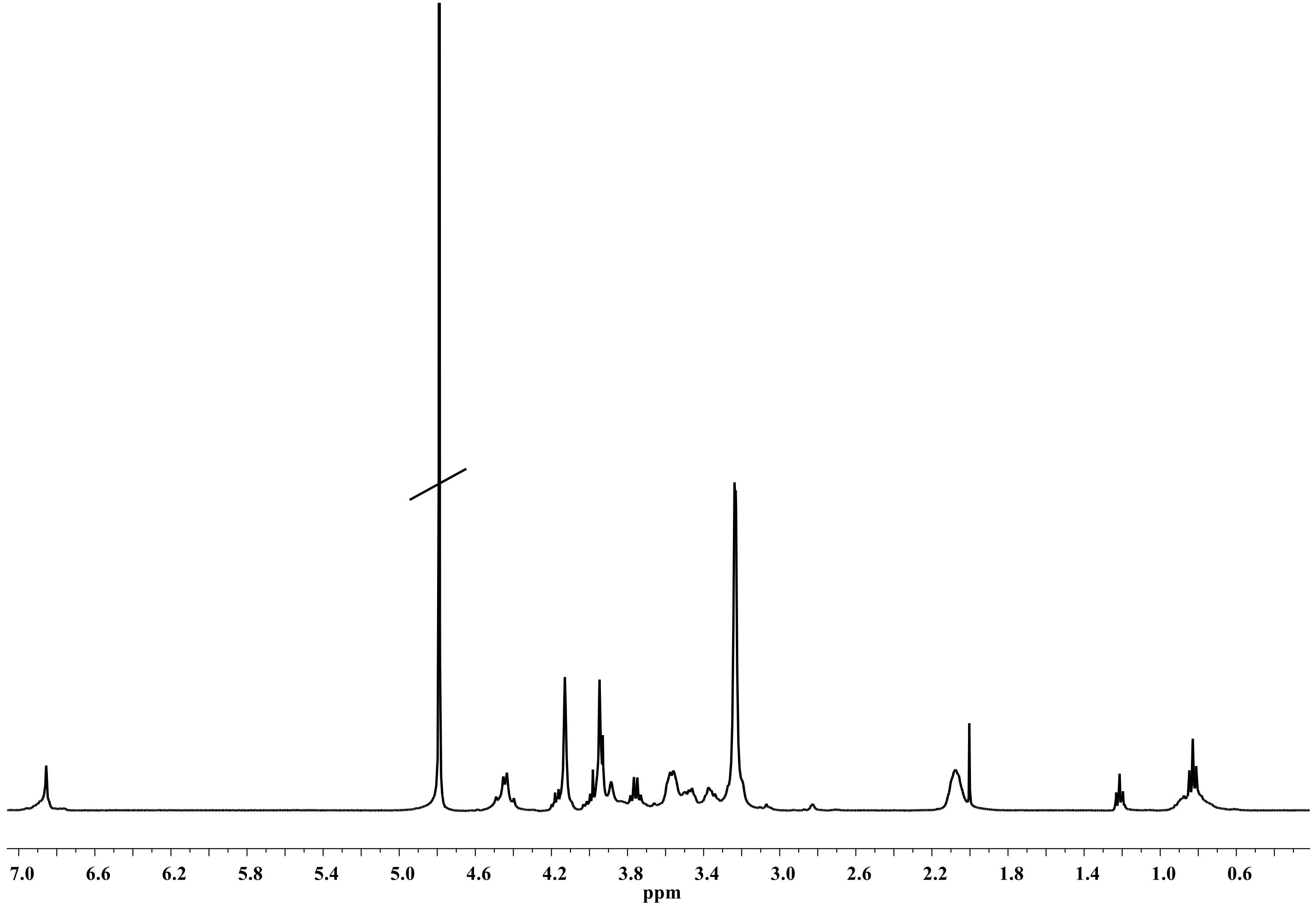

Fig. S5. **^{1}H NMR spectrum of 4,8,14,18,23,26,28,31,32,35-deca-[(*N*-(3′,3′-dimethyl-3′-{(ethoxycarbonyl [*S*-methyl]methyl)amidocarbonylmethyl}ammoniumpropyl)carbamoyl-methoxy]-pillar[5]arene decabromide *(13)*, DMSO-*d*$_6$, 298 K, 400 MHz.**

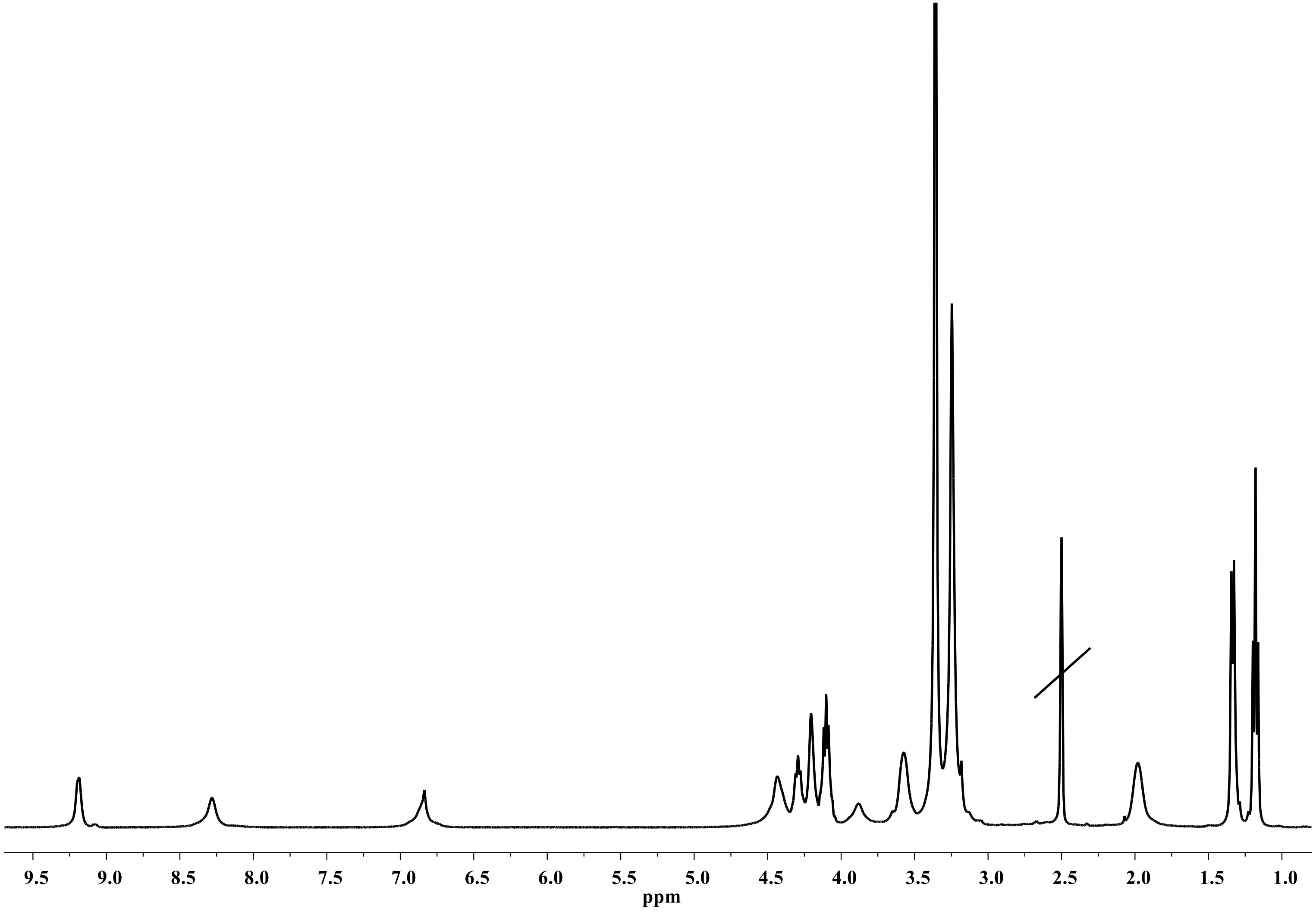

Fig. S6. ^{1}H NMR spectrum of 4,8,14,18,23,26,28,31,32,35-deca-[(*N*-(3′,3′-dimethyl-3′-{(ethoxycarbonyl [*S*-methyl]methyl)amidocarbonylmethyl}ammoniumpropyl)carbamoyl-methoxy]-pillar[5]arene decabromide *(13)*, D_2O, 298 K, 400 MHz.

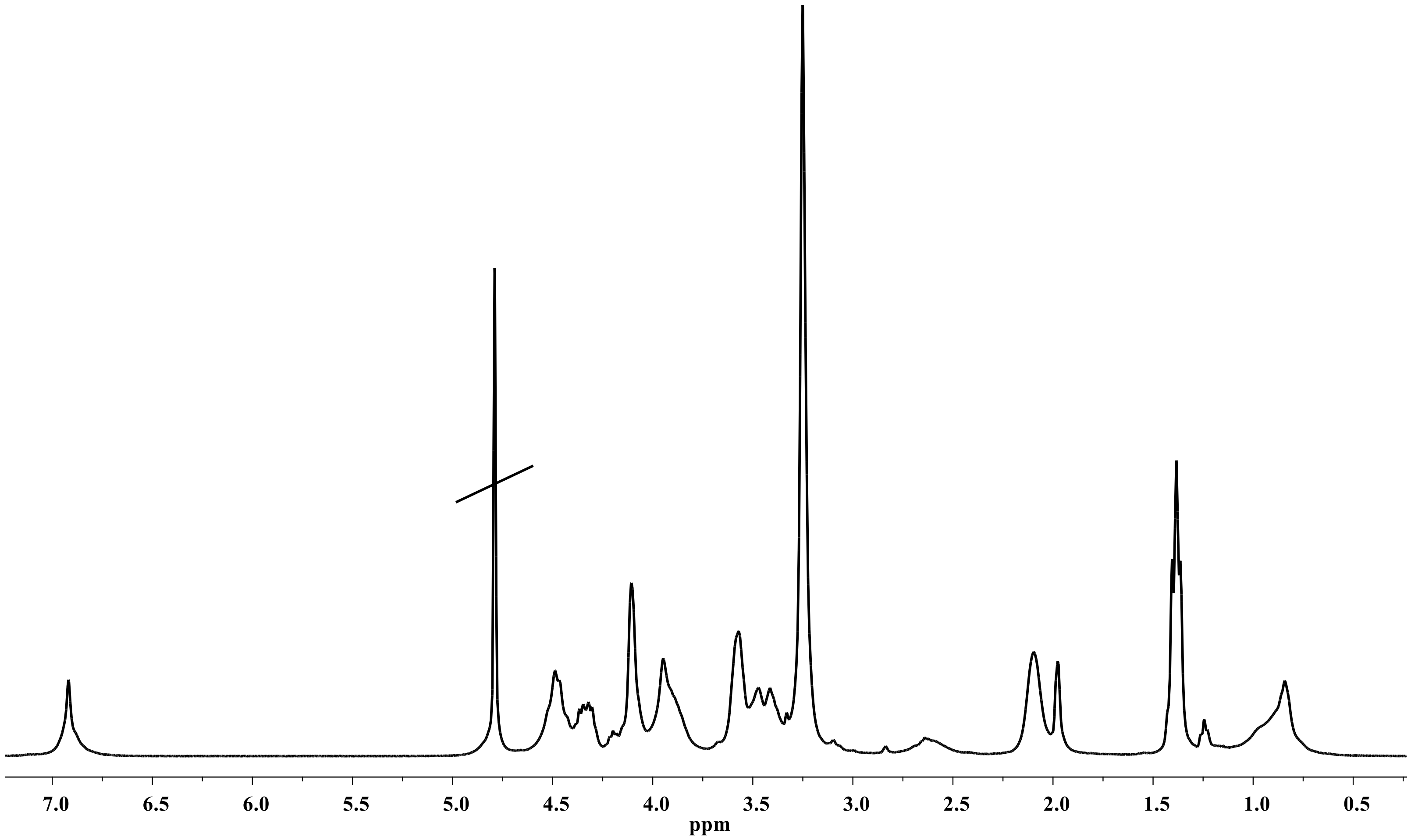

Fig. S7. **^{1}H NMR spectrum of 4,8,14,18,23,26,28,31,32,35-deca[(*N*-(3′,3′-dimethyl-3′-{([ethoxycarbonylmethyl]amidocarbonylmethyl)amidocarbonylmethyl})ammoniumpropyl)carbamoyl-methoxy]-pillar[5]arene decabromide *(14)*, DMSO-d_6, 298 K, 400 MHz.**

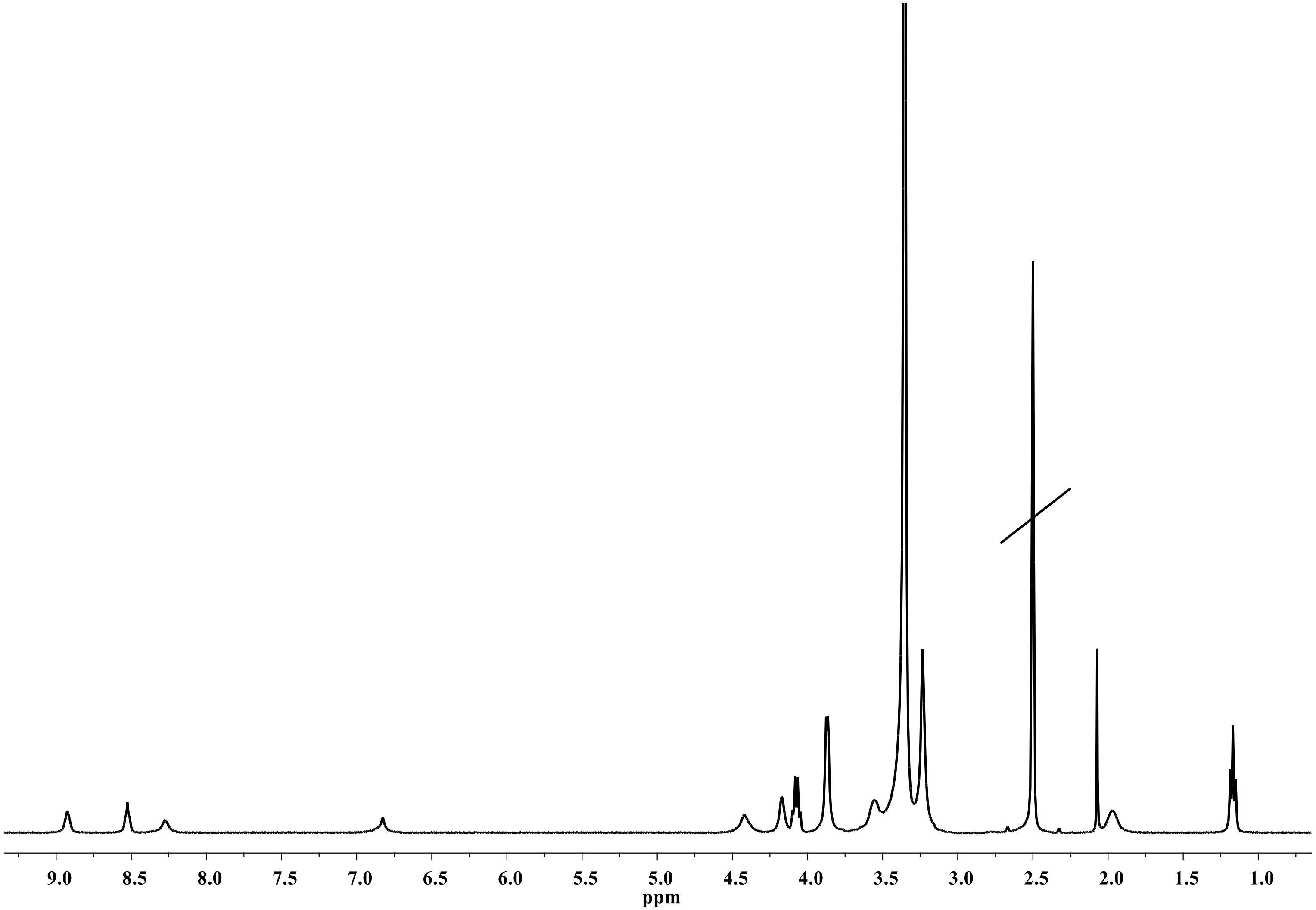

Fig. S8. **^{1}H NMR spectrum of 4,8,14,18,23,26,28,31,32,35-deca[(*N*-(3′,3′-dimethyl-3′-{([ethoxycarbonylmethyl]amidocarbonylmethyl)amidocarbonylmethyl})ammoniumpropyl)carbamoyl-methoxy]-pillar[5]arene decabromide *(14)*, D_2O, 298 K, 400 MHz.**

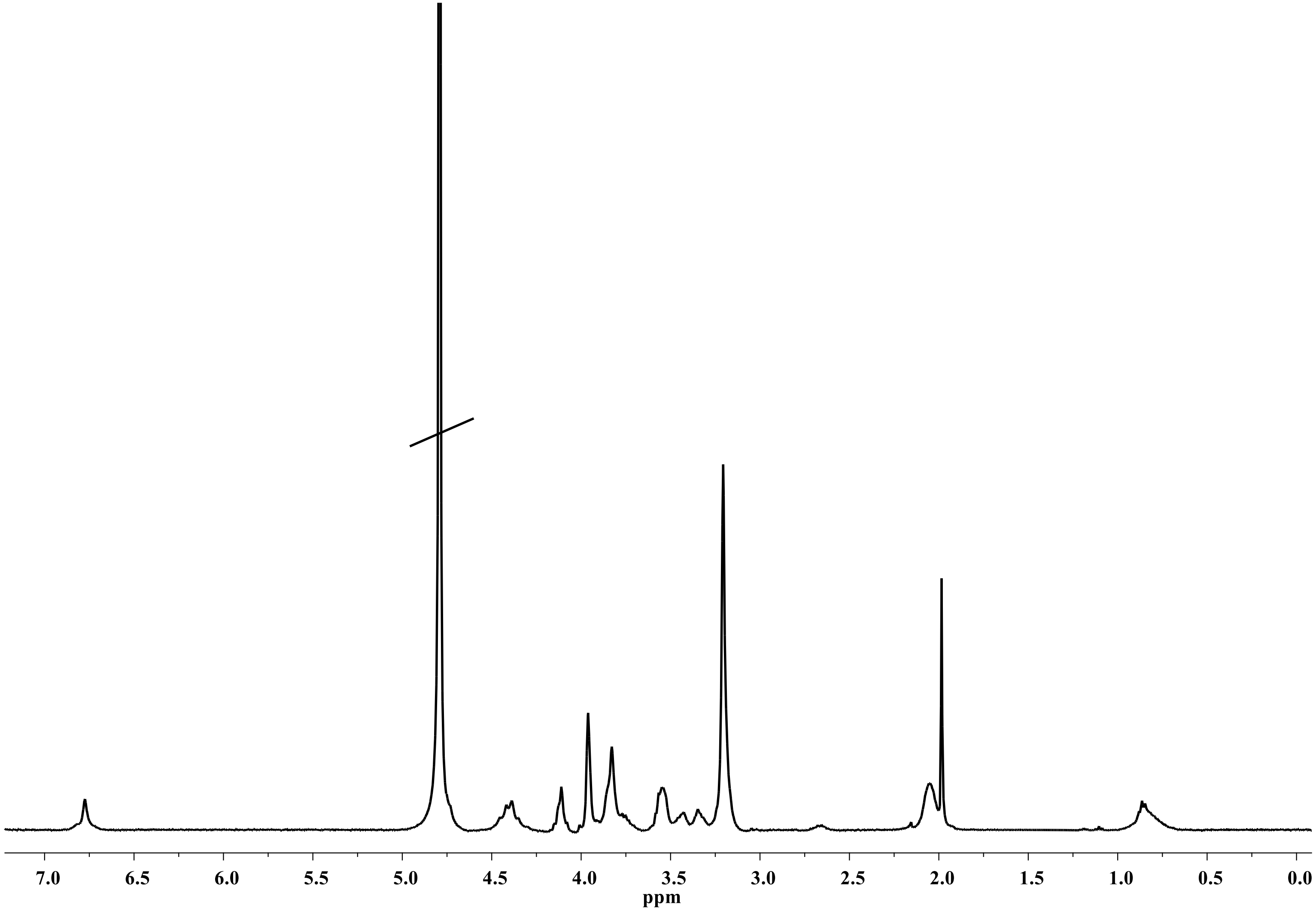

Fig. S9. ^{1}**H NMR spectrum of 4,8,14,18,23,26,28,31,32,35-deca-[(*N*-(3′,3′-dimethyl-3′-{(ethoxycarbonyl [*S*-benzyl]methyl)amidocarbonylmethyl}ammoniumpropyl)carbamoyl-methoxy]-pillar[5]arene decabromide *(15)*, DMSO-*d*$_6$, 298 K, 400 MHz.**

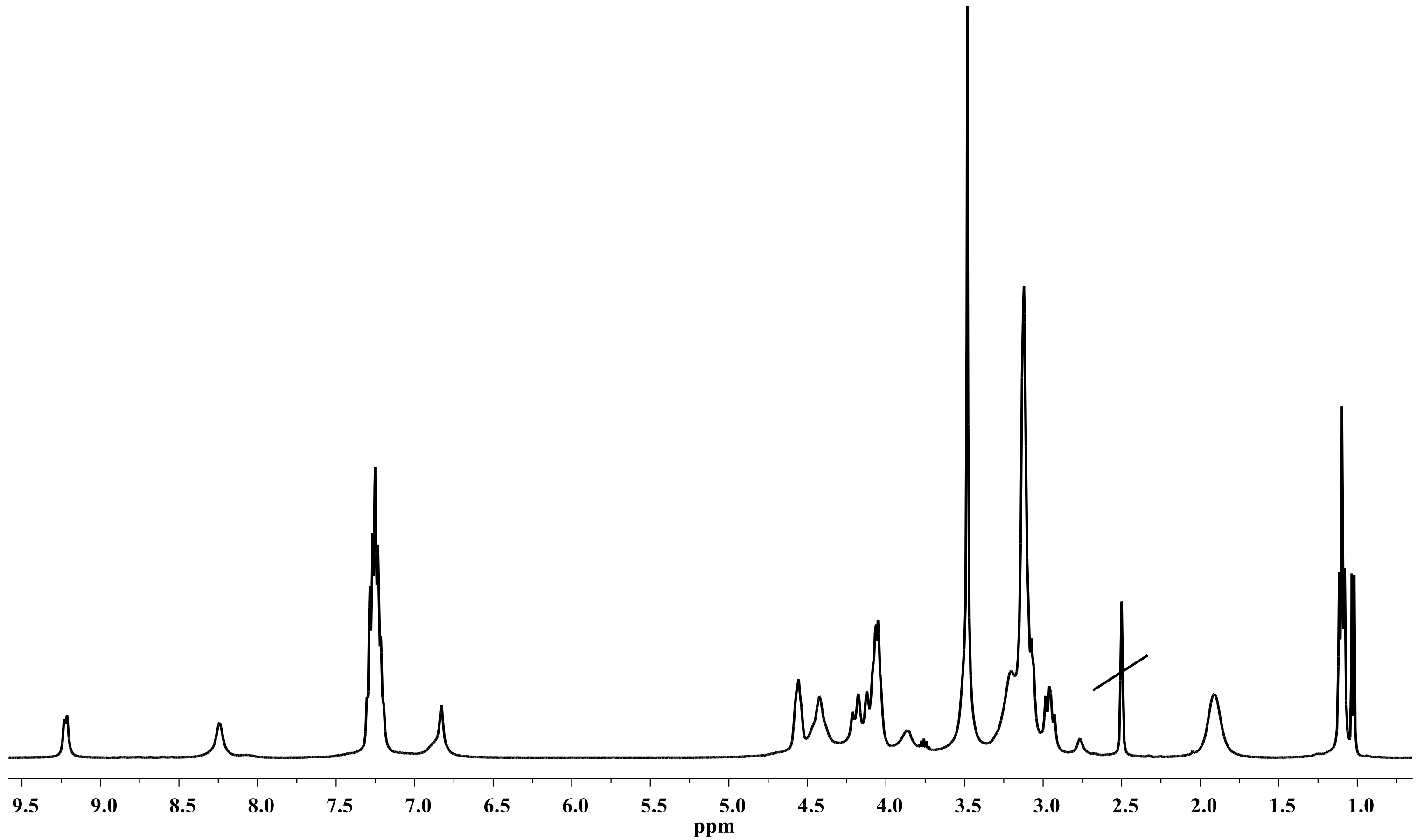

Fig. S10. ¹H NMR spectrum of 4,8,14,18,23,26,28,31,32,35-deca-[(*N*-(3′,3′-dimethyl-3′-{(ethoxycarbonyl [*S*-benzyl]methyl)amidocarbonylmethyl}ammoniumpropyl)carbamoyl-methoxy]-pillar[5]arene decabromide *(15)*, D_2O, 298 K, 400 MHz.

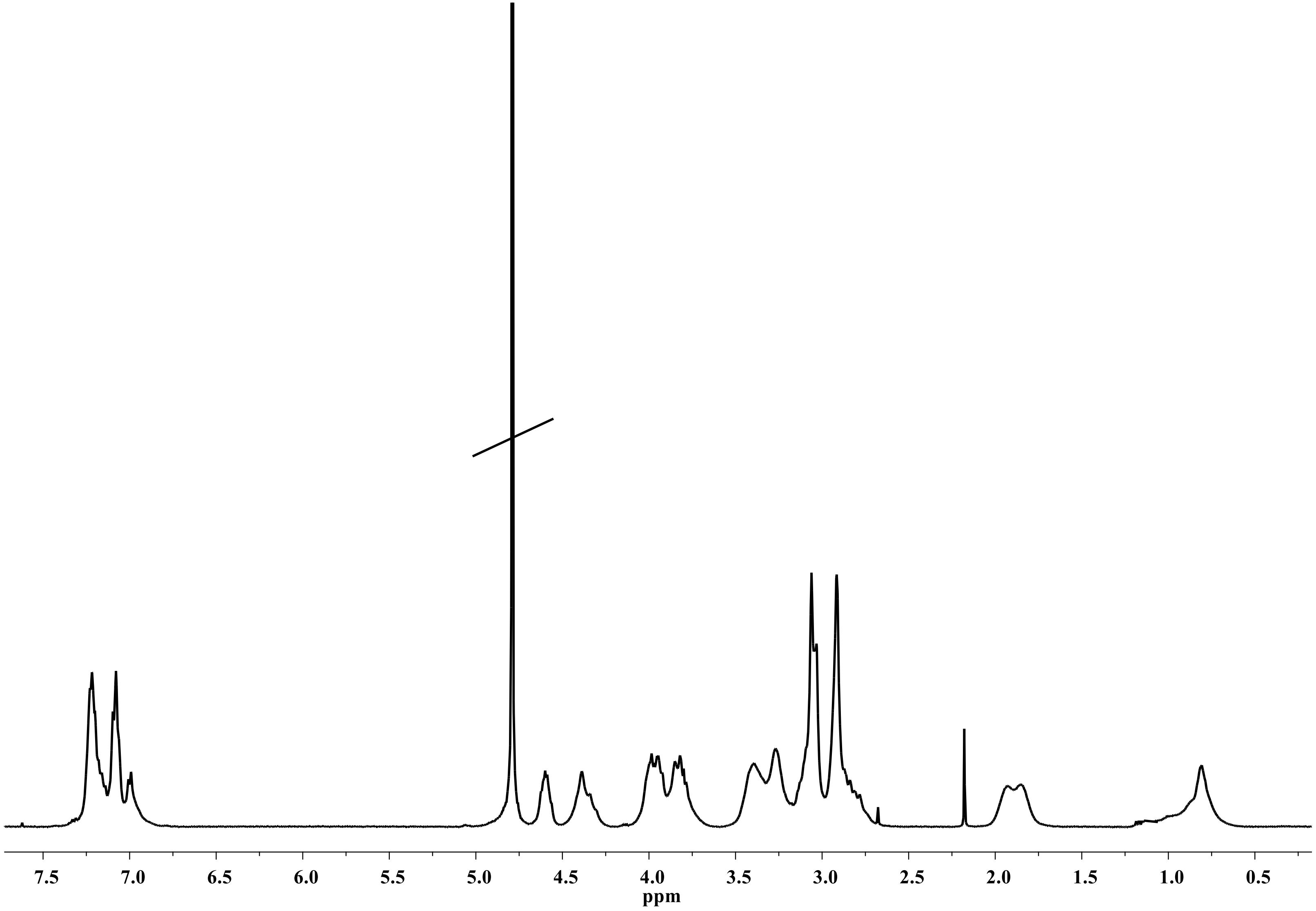

Fig. S11. ^{1}H NMR spectrum of *N*-(3-(dimethylamino)propyl)-2-(4-methoxyphenoxy)acetamide *(16)*, $CDCl_3$, 298 K, 400 MHz.

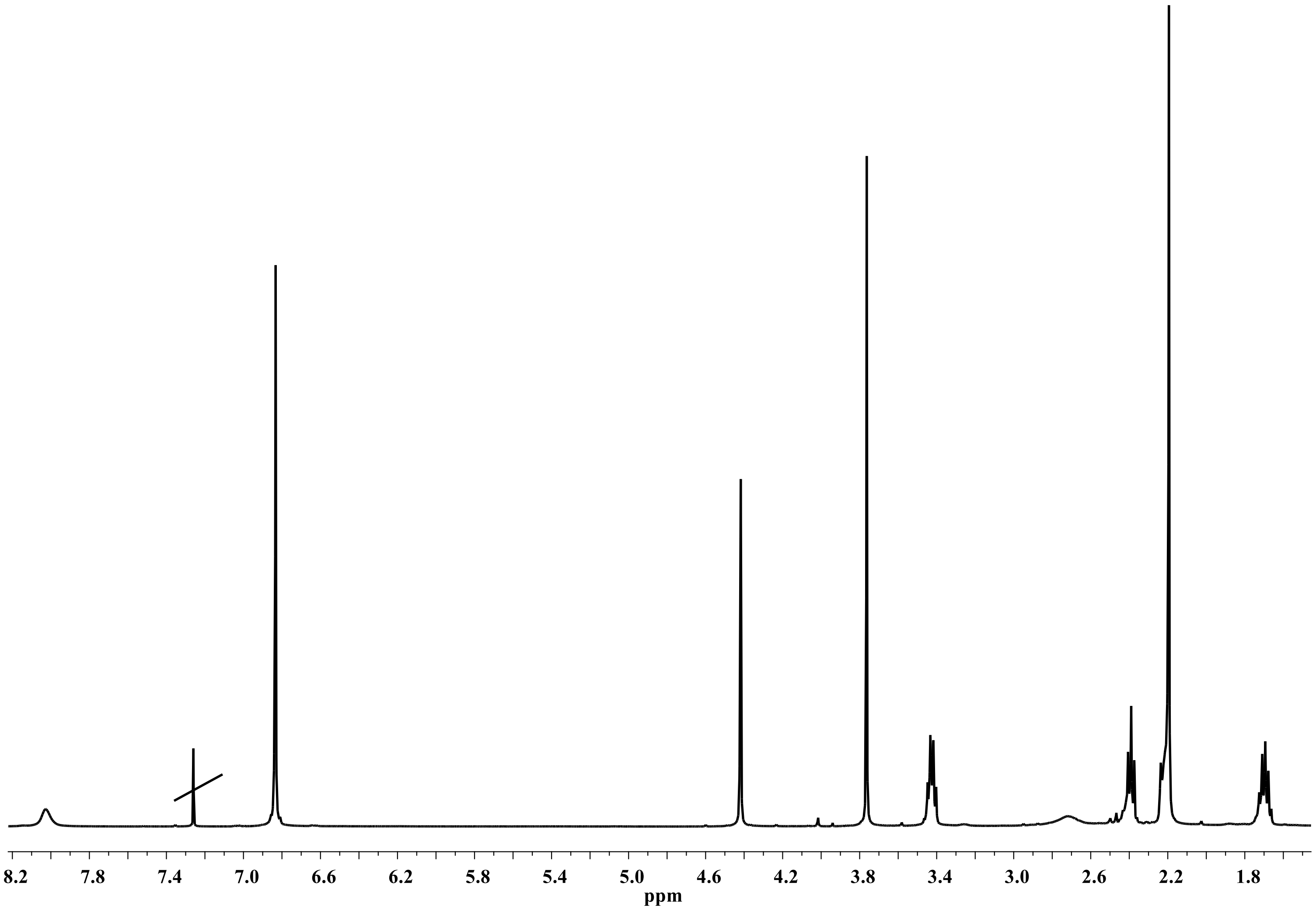

Fig. S12. ^{1}**H NMR spectrum of *N*-(2-((1-ethoxy-1-oxopropan-2-yl)amino)-2-oxoethyl)-3-(2-(4-methoxyphenoxy)acetamido)-*N*,*N*-dimethylpropan-1-ammonium bromide *(17)*, $CDCl_3$, 298 K, 400 MHz.**

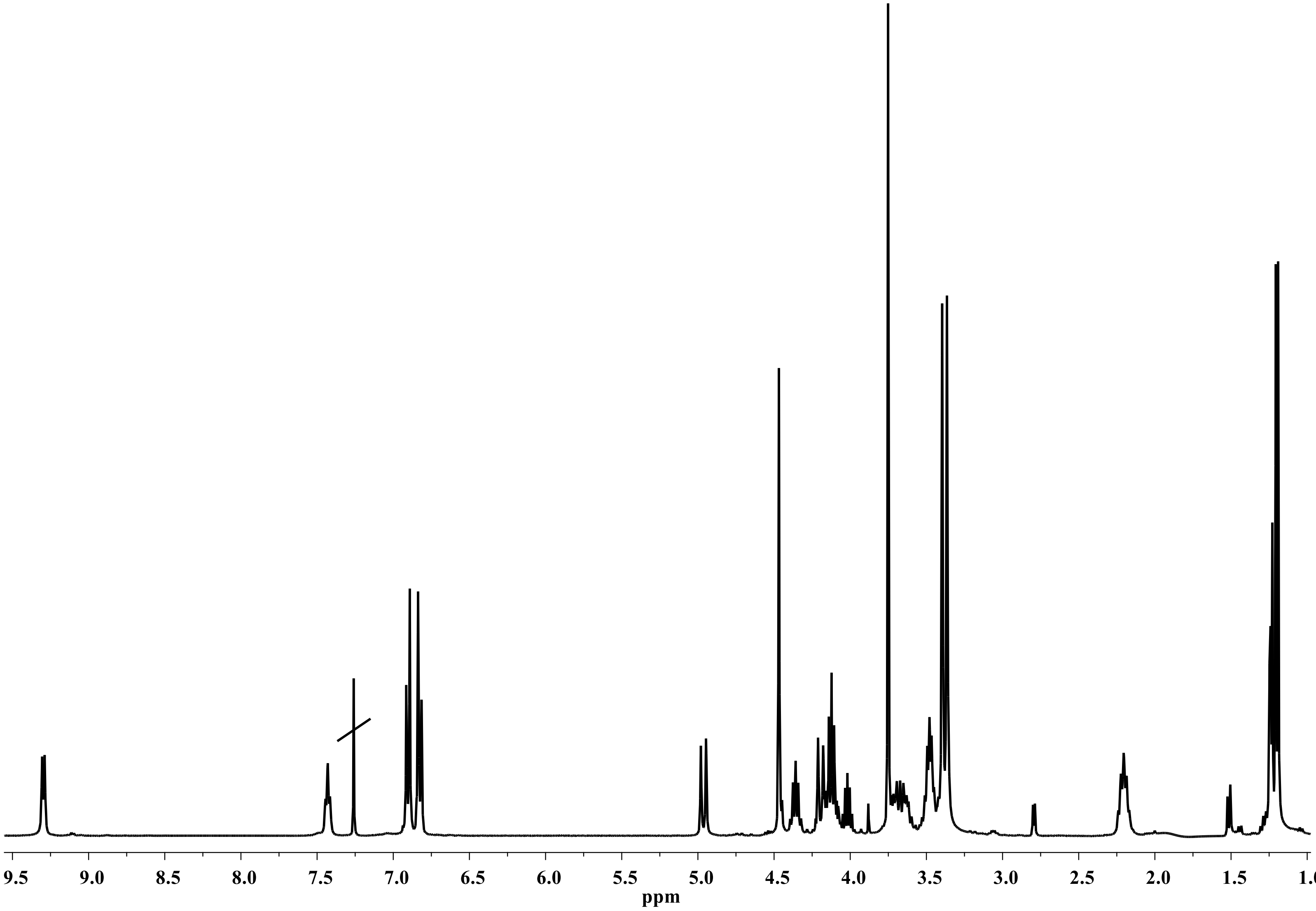

Fig. S13. **^{1}H NMR spectrum of *N*-(2-((1-ethoxy-1-oxo-3-phenylpropan-2-yl)amino)-2-oxoethyl)-3-(2-(4-methoxyphenoxy)acetamido)-*N,N*-dimethylpropan-1-ammonium bromide *(18)*, $CDCl_3$, 298 K, 400 MHz.**

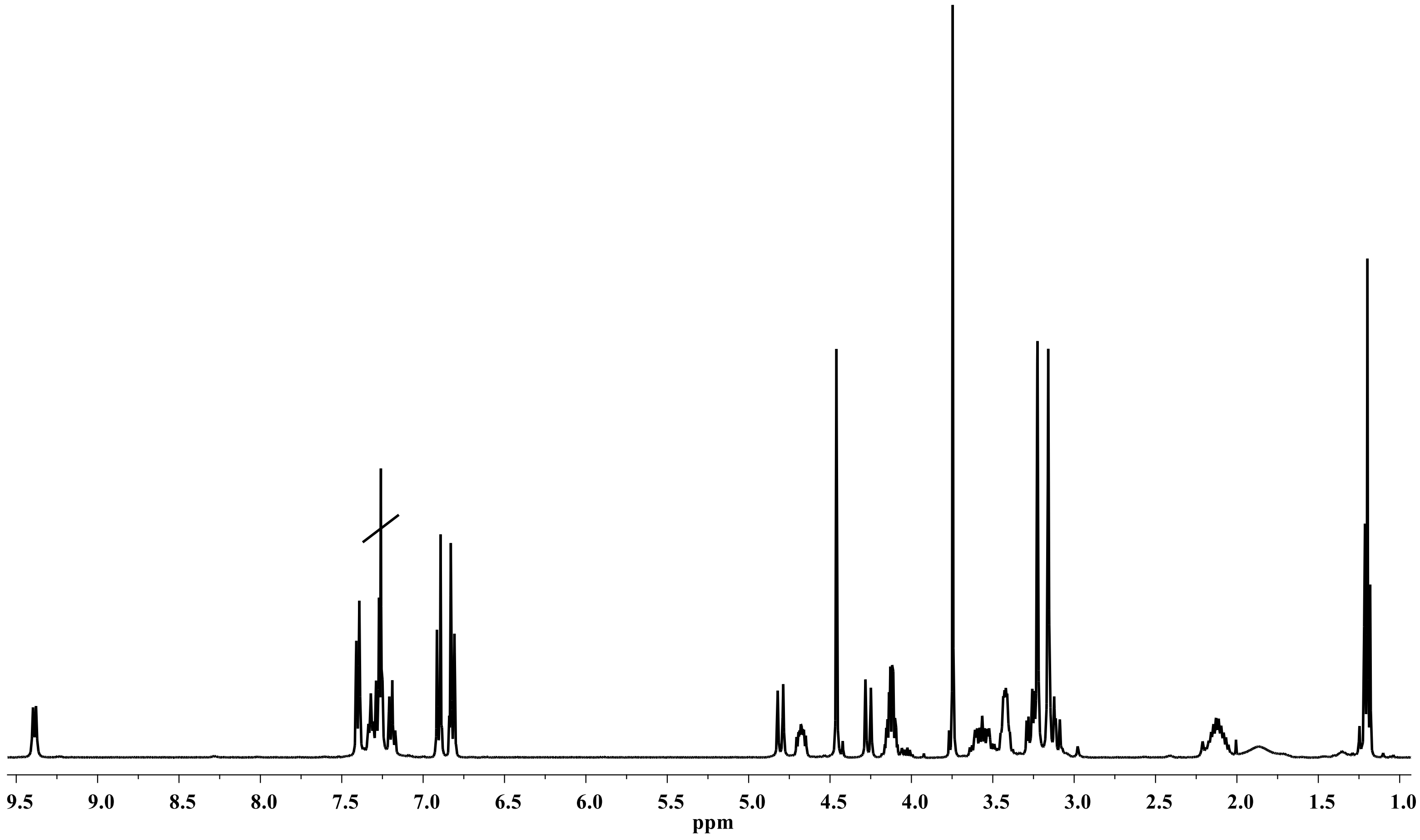

Fig. S14. ^{13}C NMR spectrum of 4,8,14,18,23,26,28,31,32,35-deca-[(*N*-(3′,3′-dimethyl-3′-{(ethoxycarbonylmethyl)amidocarbonylmethyl}ammoniumpropyl)carbomoyl-methoxy]-pillar[5]arene decabromide *(12)*, DMSO-*d*$_6$, 298 K, 100 MHz.

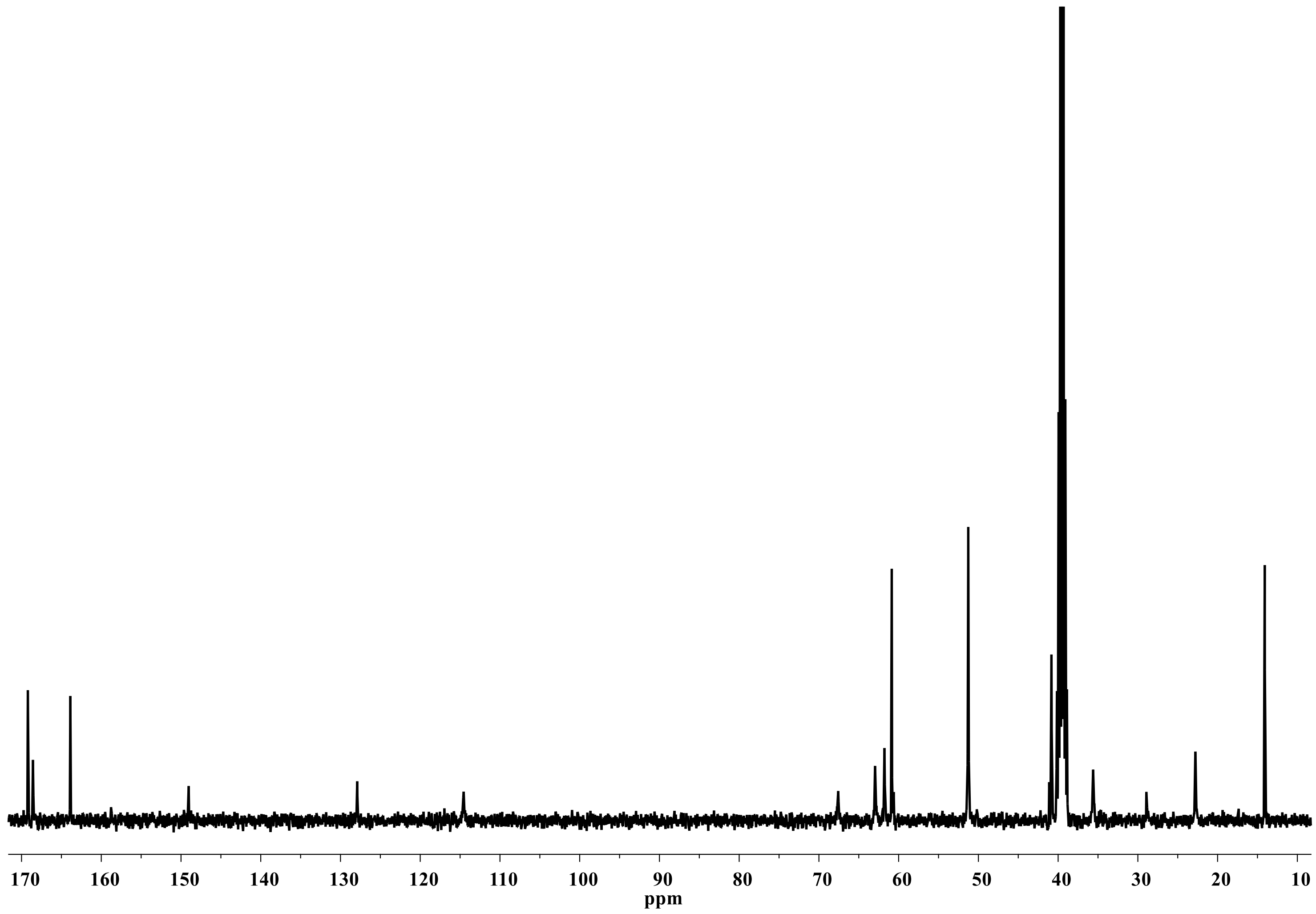

Fig. S15. ^{13}**C NMR spectrum of 4,8,14,18,23,26,28,31,32,35-deca-[(*N*-(3′,3′-dimethyl-3′-{(ethoxycarbonyl[*S*-methyl]methyl)amidocarbonylmethyl}ammoniumpropyl)carbamoyl-methoxy]-pillar[5]arene decabromide *(13)*, DMSO-d_6, 298 K, 100 MHz.**

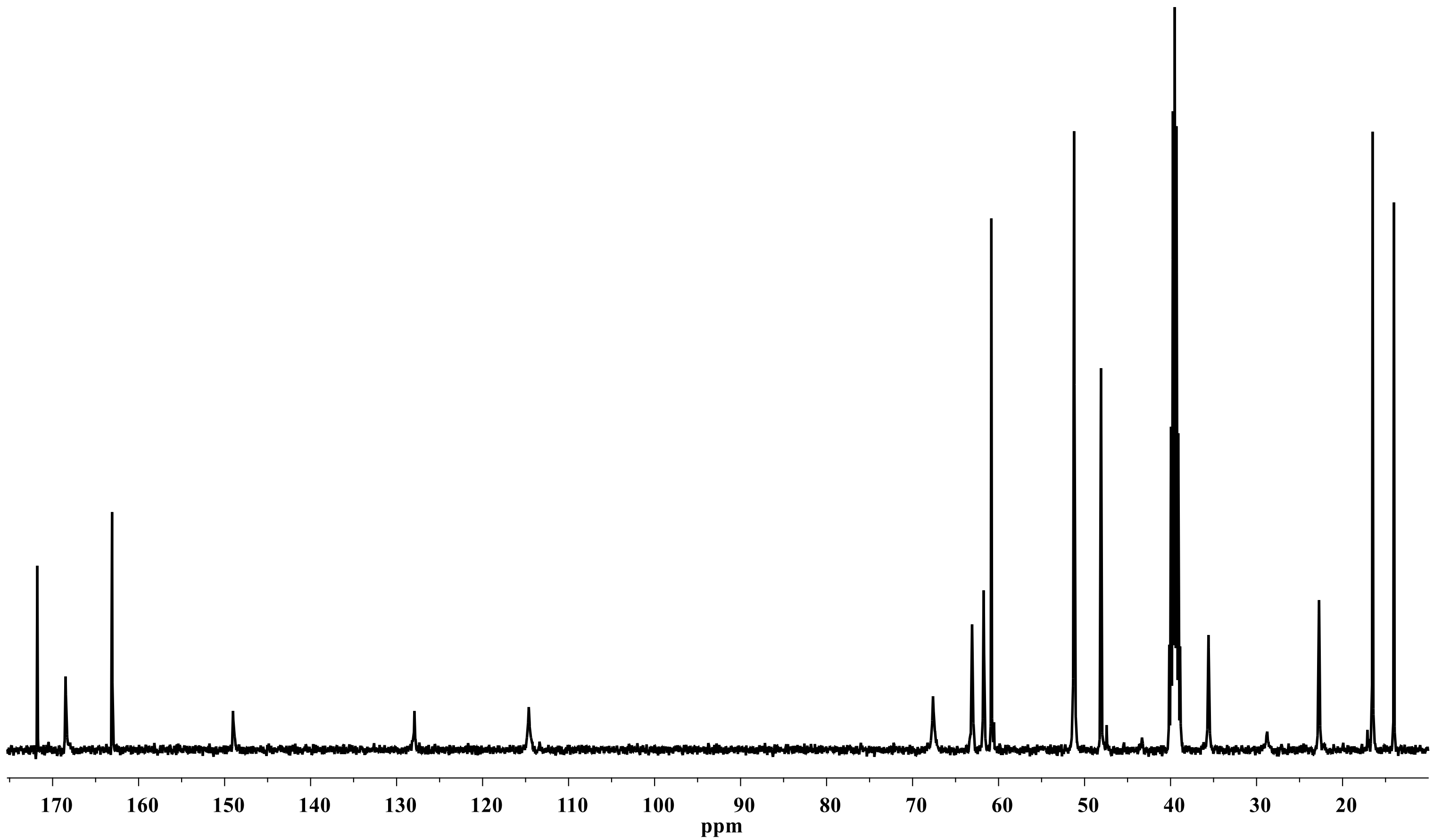

Fig. S16. ^{13}**C NMR spectrum of 4,8,14,18,23,26,28,31,32,35-deca[(*N*-(3′,3′-dimethyl-3′-{([ethoxycarbonylmethyl]amidocarbonylmethyl)amidocarbonylmethyl})ammoniumpropyl)carbamoyl-methoxy]-pillar[5]arene decabromide *(14)*, DMSO-*d*$_6$, 298 K, 100 MHz.**

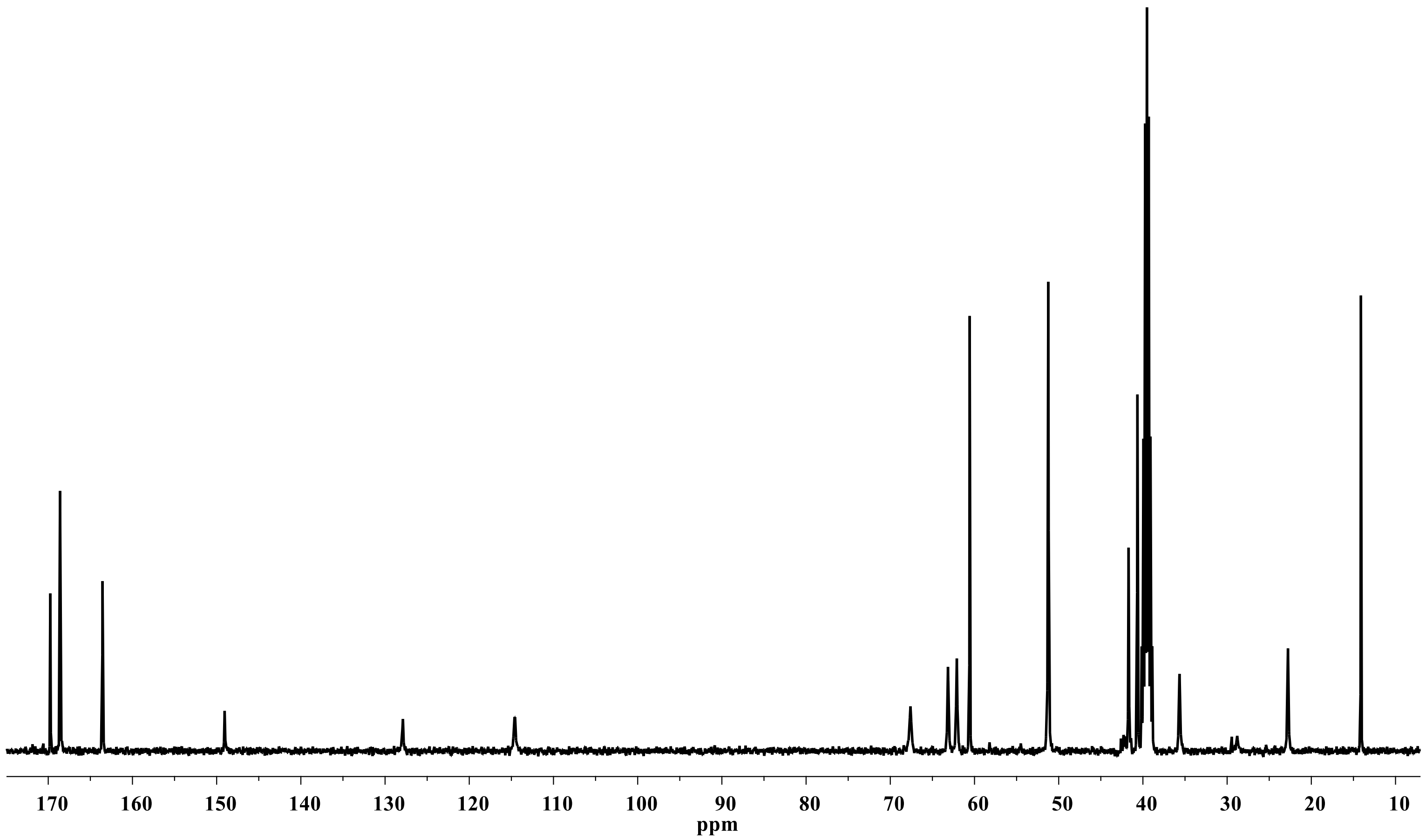

Fig. S17. ^{13}**C NMR spectrum of 4,8,14,18,23,26,28,31,32,35-deca-[(*N*-(3′,3′-dimethyl-3′-{(ethoxycarbonyl [*S*-benzyl]methyl)amidocarbonylmethyl}ammoniumpropyl)carbamoyl-methoxy]-pillar[5]arene decabromide *(15)*, DMSO-d_6, 298 K, 100 MHz.**

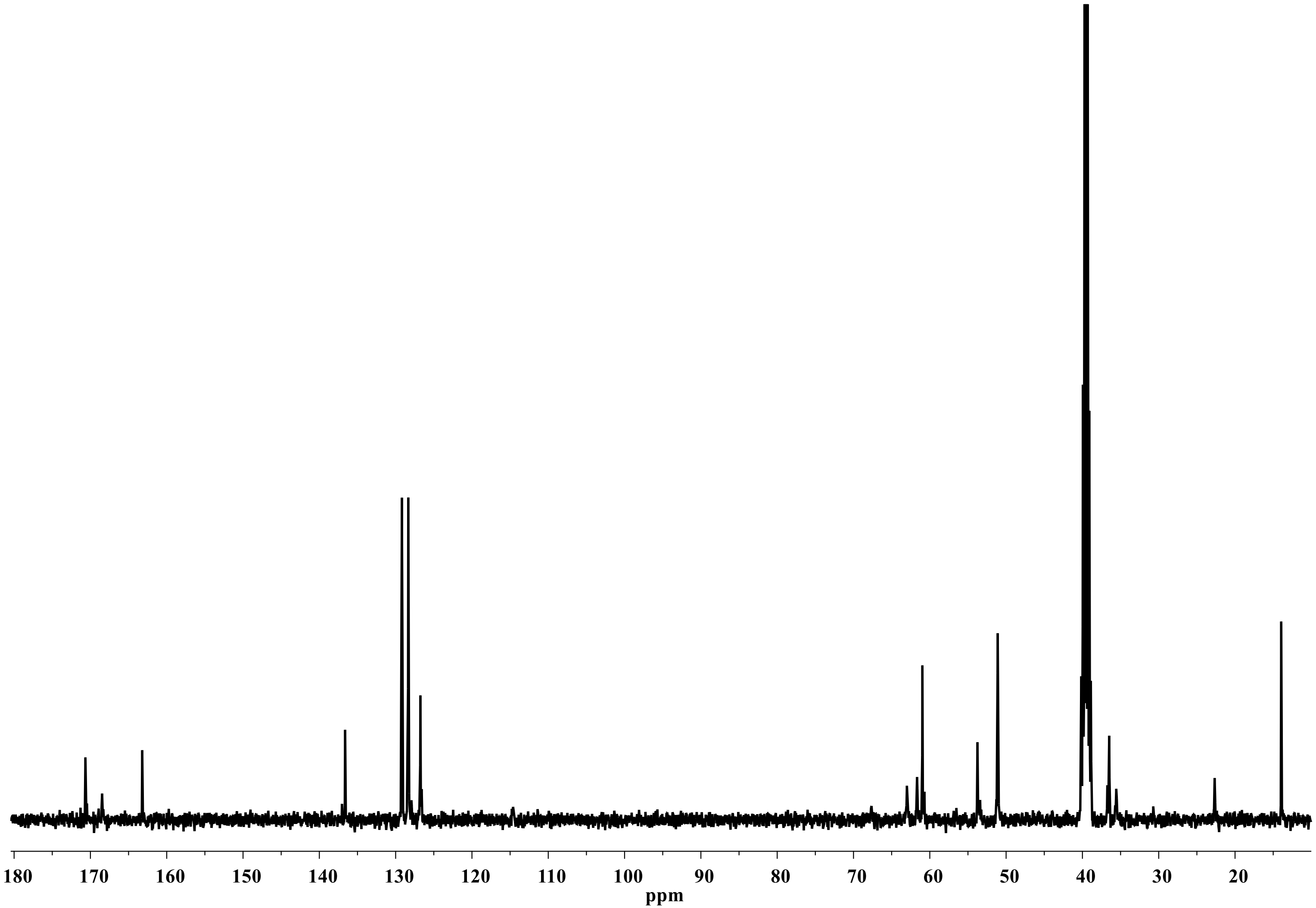

Fig. S18. [1]**H NMR spectrum of *N*-(2-((1-ethoxy-1-oxopropan-2-yl)amino)-2-oxoethyl)-3-(2-(4-methoxyphenoxy)acetamido)-*N*,*N*-dimethylpropan-1-ammonium bromide *(17)*, $CDCl_3$, 298 K, 100 MHz.**

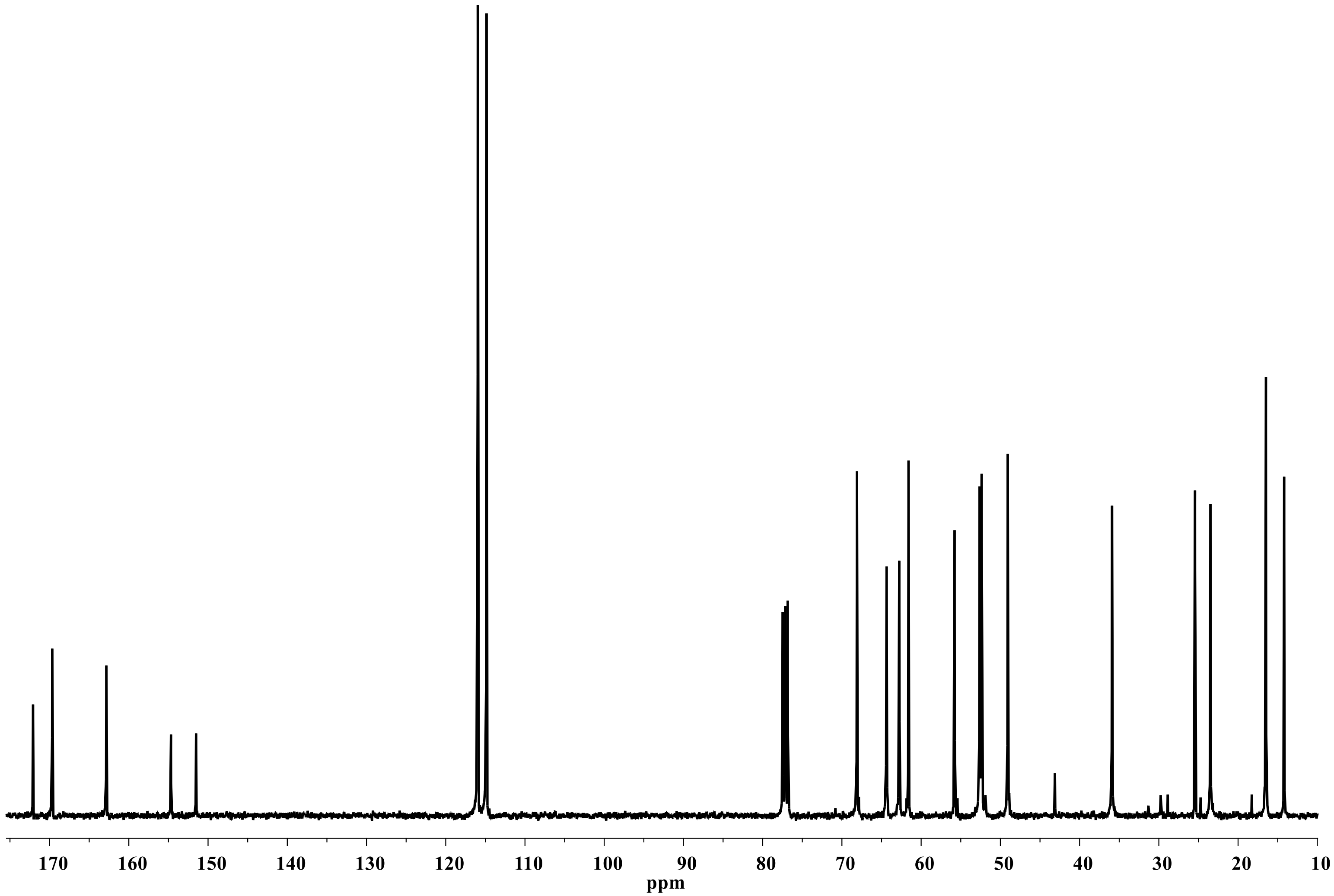

Fig. S19. ^{1}H NMR spectrum of *N*-(2-((1-ethoxy-1-oxo-3-phenylpropan-2-yl)amino)-2-oxoethyl)-3-(2-(4-methoxyphenoxy)acetamido)-*N,N*-dimethylpropan-1-ammonium bromide *(18)*, $CDCl_3$, 298 K, 100 MHz.

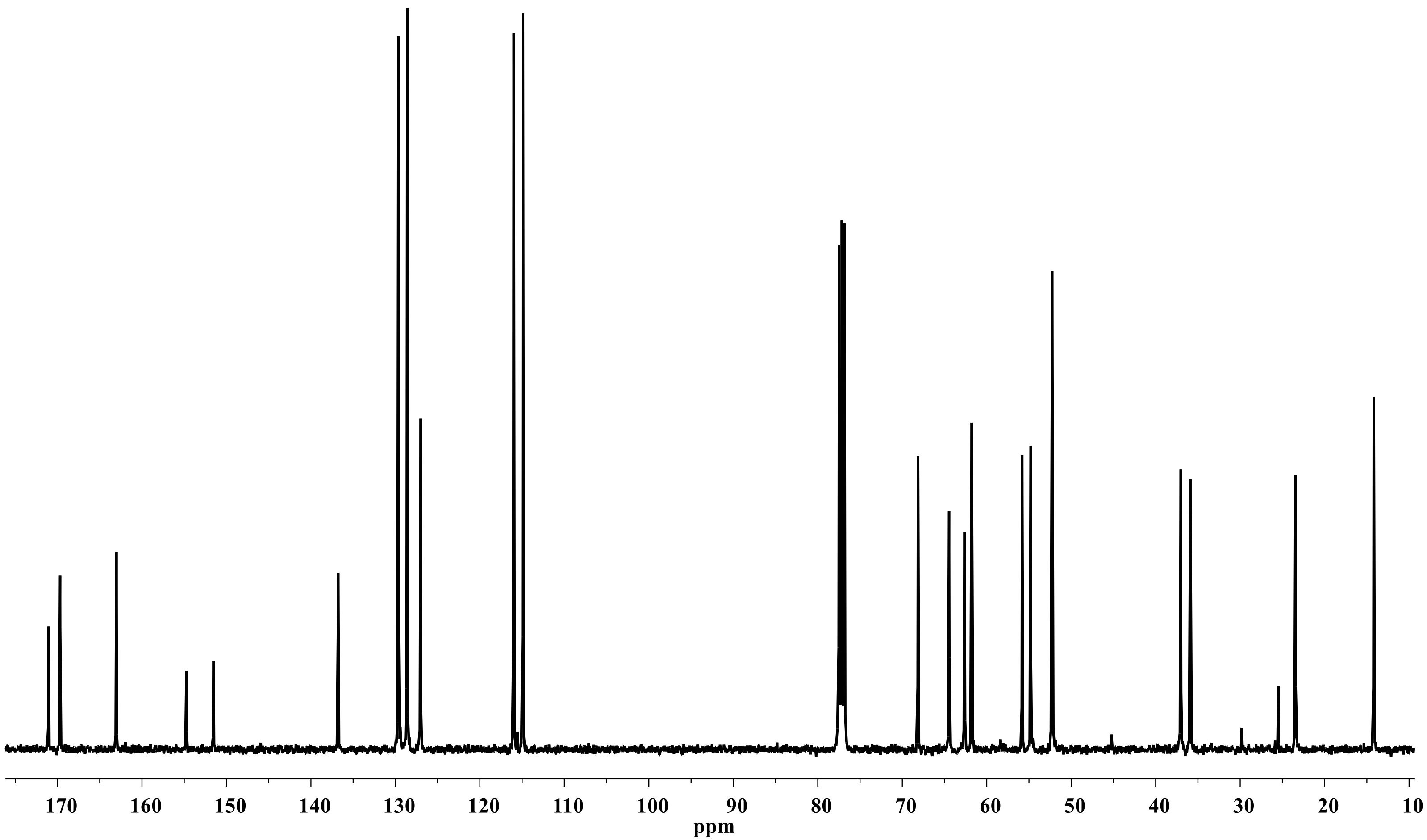

Fig. S20. Mass spectrum (ESI) of 4,8,14,18,23,26,28,31,32,35-deca-[(*N*-(3′,3′-dimethyl-3′-{(ethoxycarbonylmethyl)amidocarbonylmethyl}ammoniumpropyl)carbomoyl-methoxy]-pillar[5]arene decabromide *(12)*.

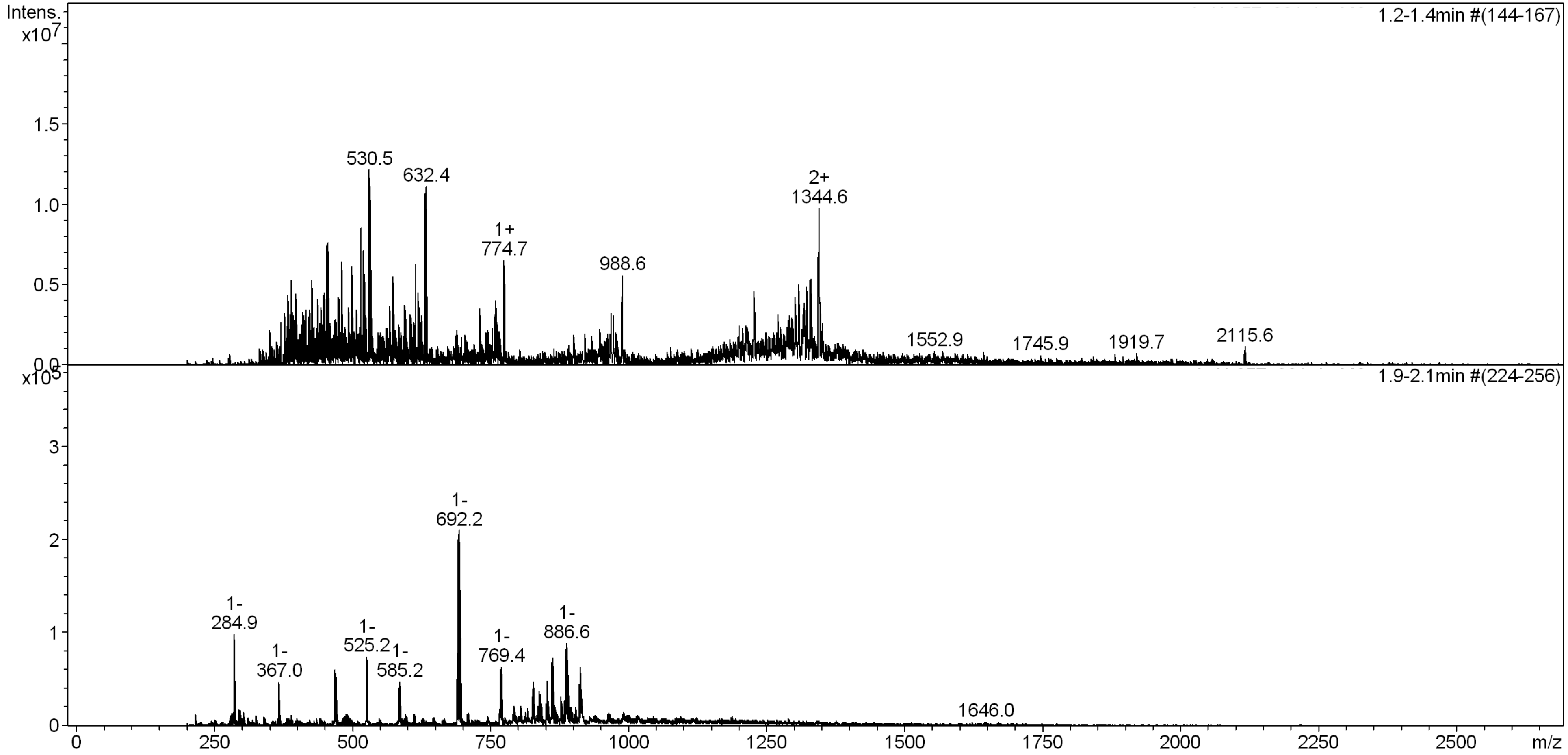

Fig. S21. Mass spectrum (ESI) of 4,8,14,18,23,26,28,31,32,35-deca-[(*N*-(3′,3′-dimethyl-3′-{(ethoxycarbonyl [*S*-methyl]methyl)amidocarbonylmethyl}ammoniumpropyl)carbamoyl-methoxy]-pillar[5]arene decabromide *(13)*.

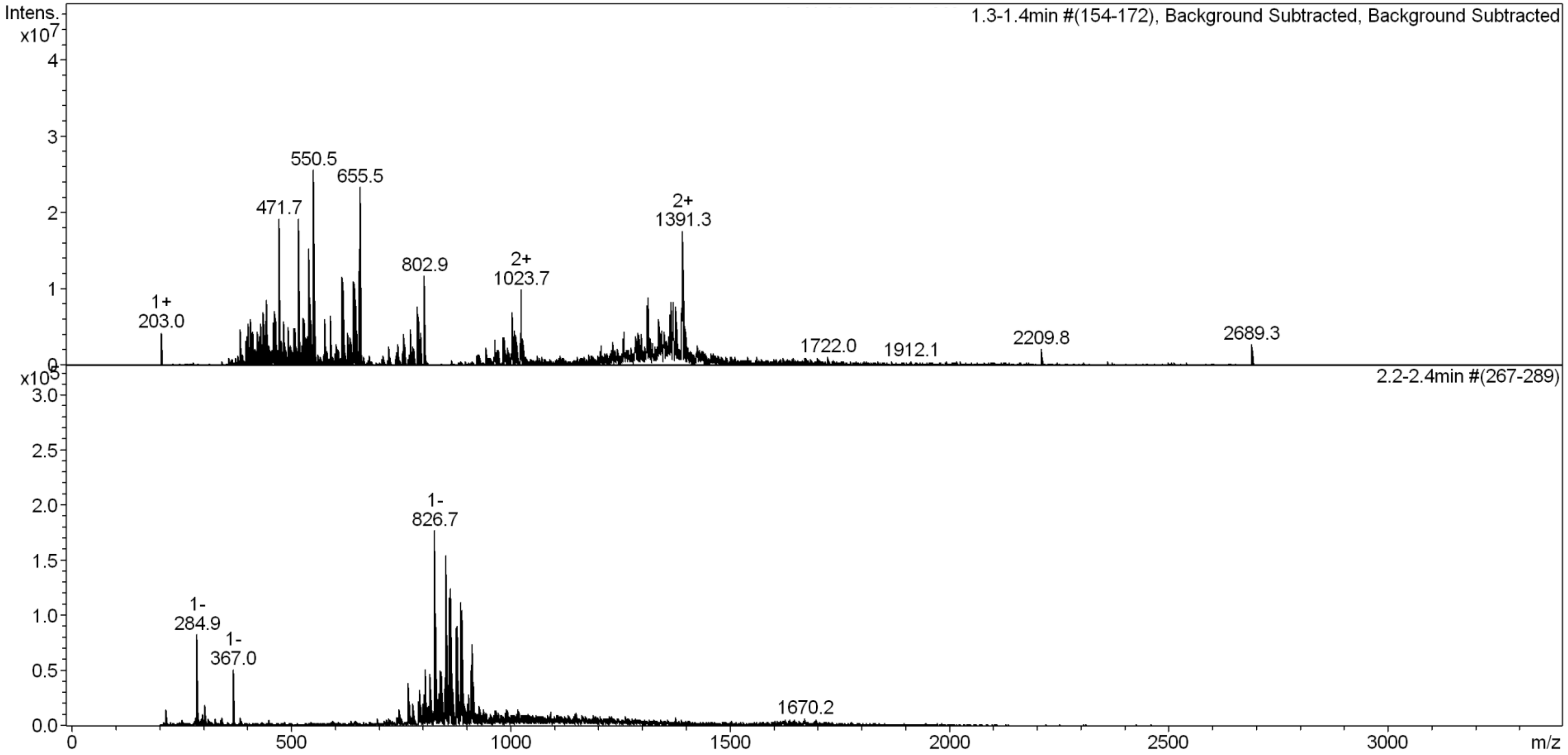

Fig. S22. Mass spectrum (ESI) of 4,8,14,18,23,26,28,31,32,35-deca[(*N*-(3′,3′-dimethyl-3′-{([ethoxycarbonylmethyl]amidocarbonylmethyl)amidocarbonylmethyl})ammoniumpropyl)carbamoyl-methoxy]-pillar[5]arene decabromide *(14)*.

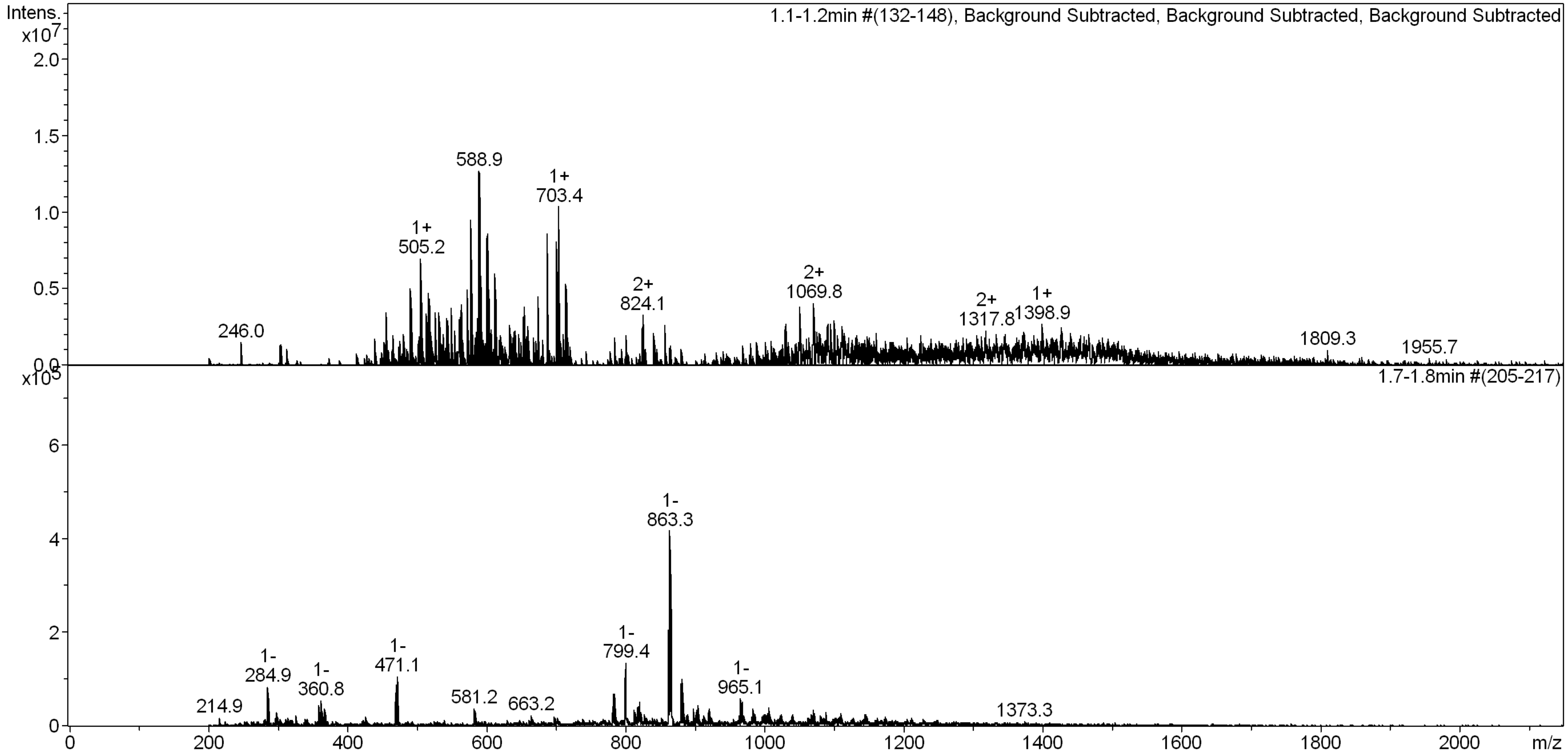

Fig. S23. Mass spectrum (ESI) of 4,8,14,18,23,26,28,31,32,35-deca-[(*N*-(3′,3′-dimethyl-3′-{(ethoxycarbonyl [*S*-benzyl]methyl)amidocarbonylmethyl}ammoniumpropyl)carbamoyl-methoxy]-pillar[5]arene decabromide *(15)*.

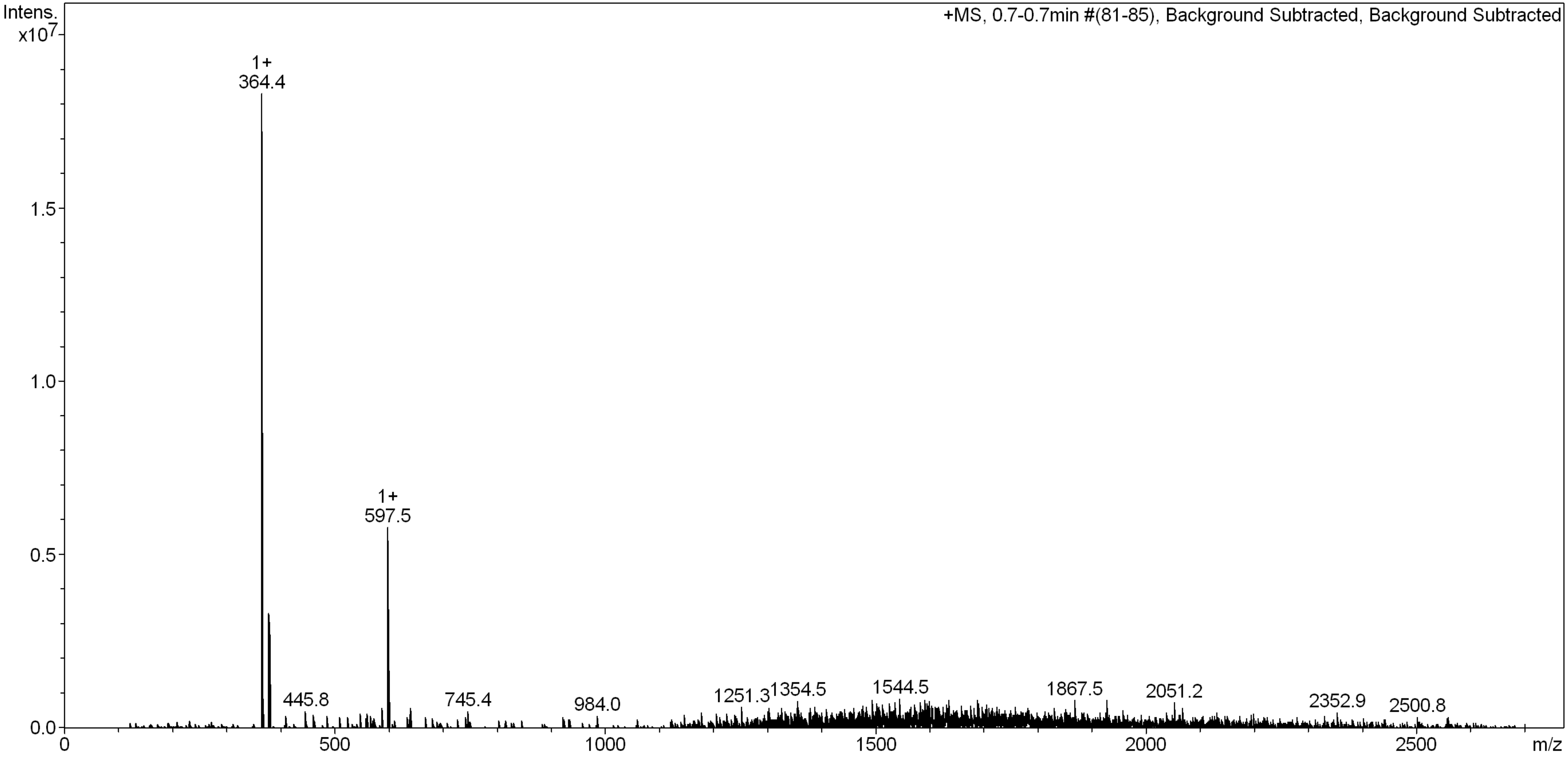

Fig. S24. Mass spectrum (ESI) of *N*-(2-((1-ethoxy-1-oxopropan-2-yl)amino)-2-oxoethyl)-3-(2-(4-methoxyphenoxy)acetamido)-*N,N*-dimethylpropan-1-ammonium bromide *(17)*.

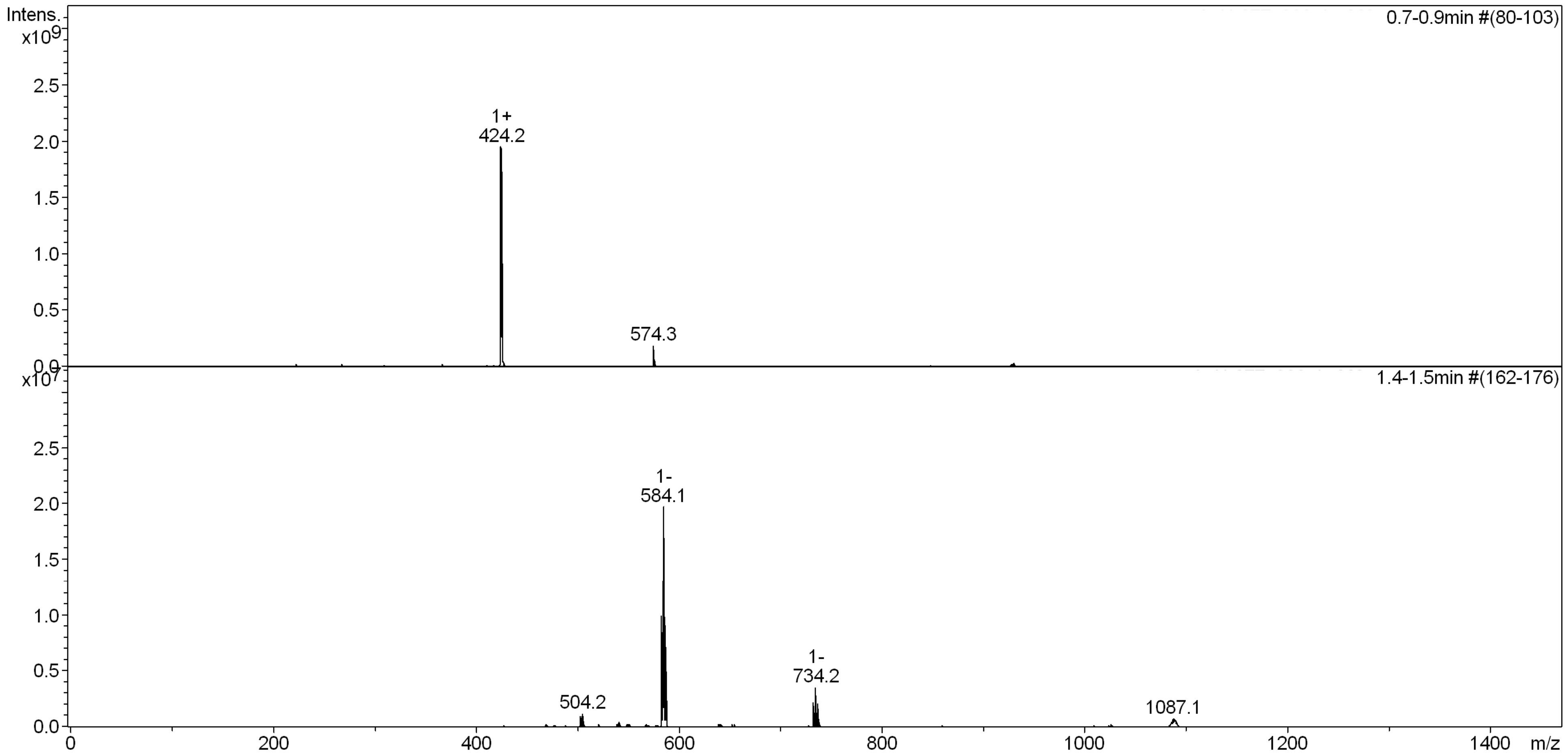

Fig. S25. Mass spectrum (ESI) of *N*-(2-((1-ethoxy-1-oxo-3-phenylpropan-2-yl)amino)-2-oxoethyl)-3-(2-(4-methoxyphenoxy)acetamido)-*N,N*-dimethylpropan-1-ammonium bromide *(18)*.

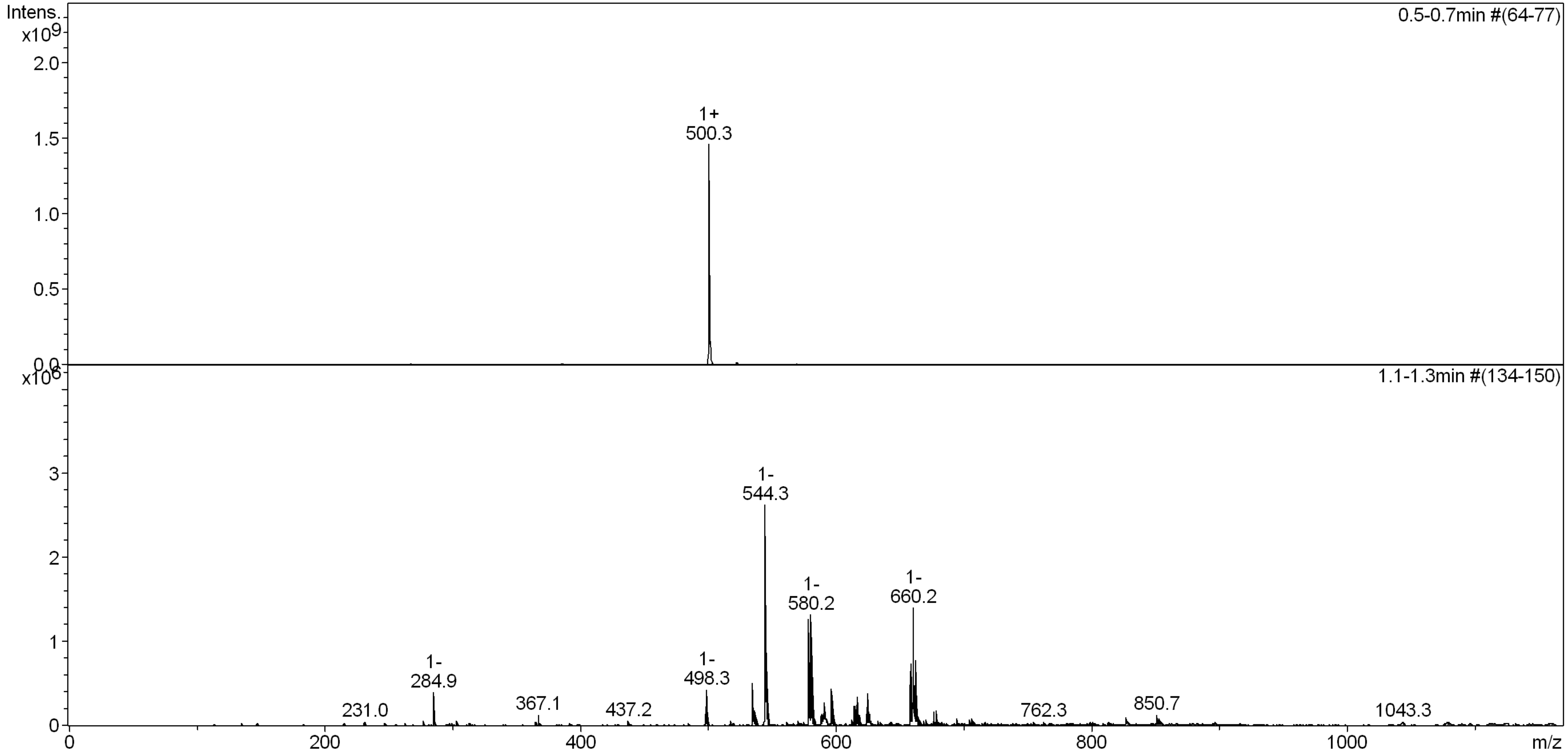

Fig. S26. IR spectrum of 4,8,14,18,23,26,28,31,32,35-deca-[(*N*-(3′,3′-dimethyl-3′-{(ethoxycarbonylmethyl)amidocarbonylmethyl}ammoniumpropyl)carbomoyl-methoxy]-pillar[5]arene decabromide *(12)*.

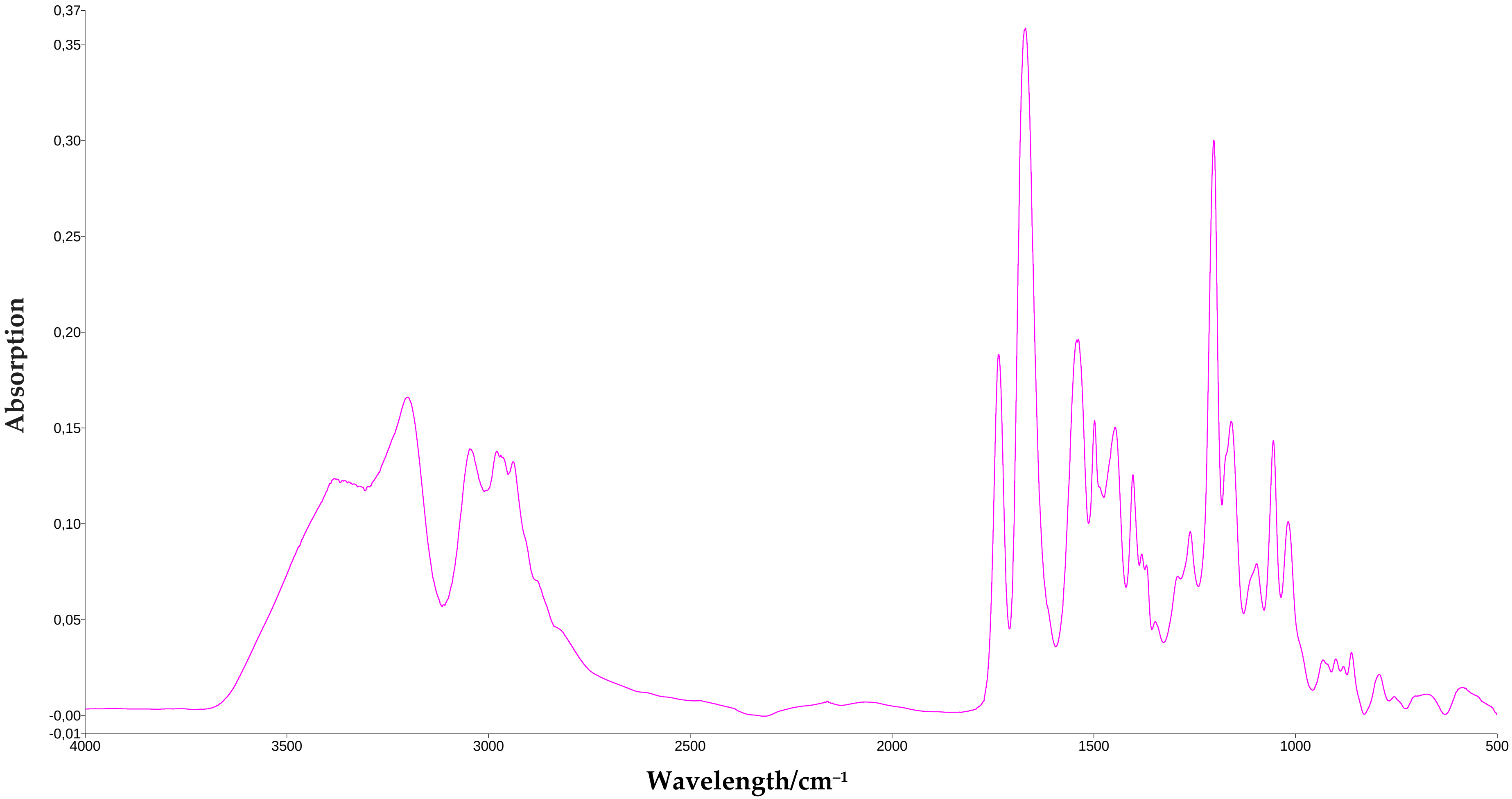

Fig. S27. IR spectrum of 4,8,14,18,23,26,28,31,32,35-deca-[(*N*-(3′,3′-dimethyl-3′-{(ethoxycarbonyl[*S*-methyl]methyl)amidocarbonylmethyl}ammoniumpropyl)carbamoyl-methoxy]-pillar[5]arene decabromide *(13)*.

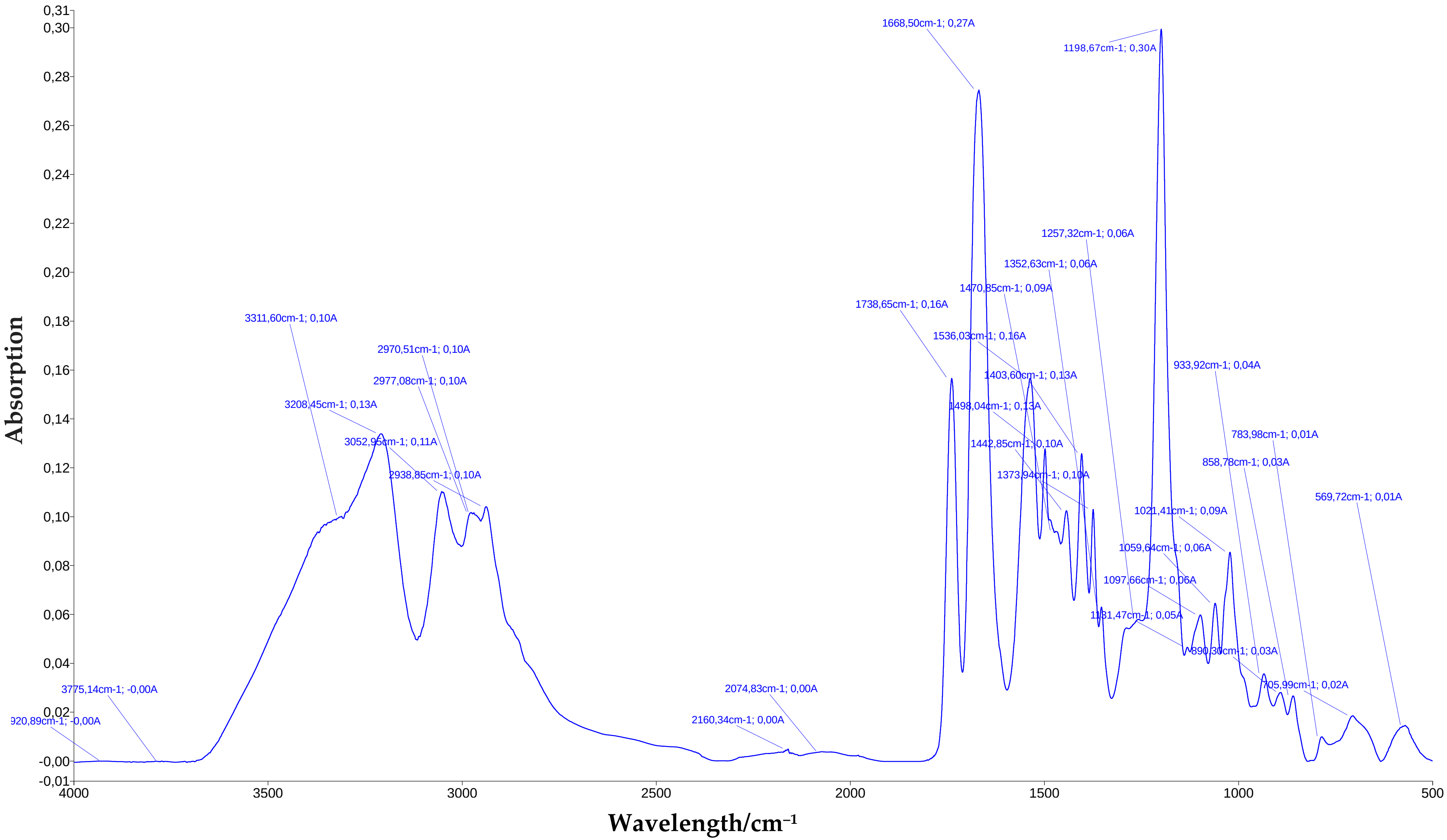

Fig. S28. IR spectrum of 4,8,14,18,23,26,28,31,32,35-deca[(*N*-(3′,3′-dimethyl-3′-{([ethoxycarbonylmethyl]amidocarbonylmethyl)amidocarbonylmethyl})ammoniumpropyl)carbamoyl-methoxy]-pillar[5]arene decabromide *(14)*.

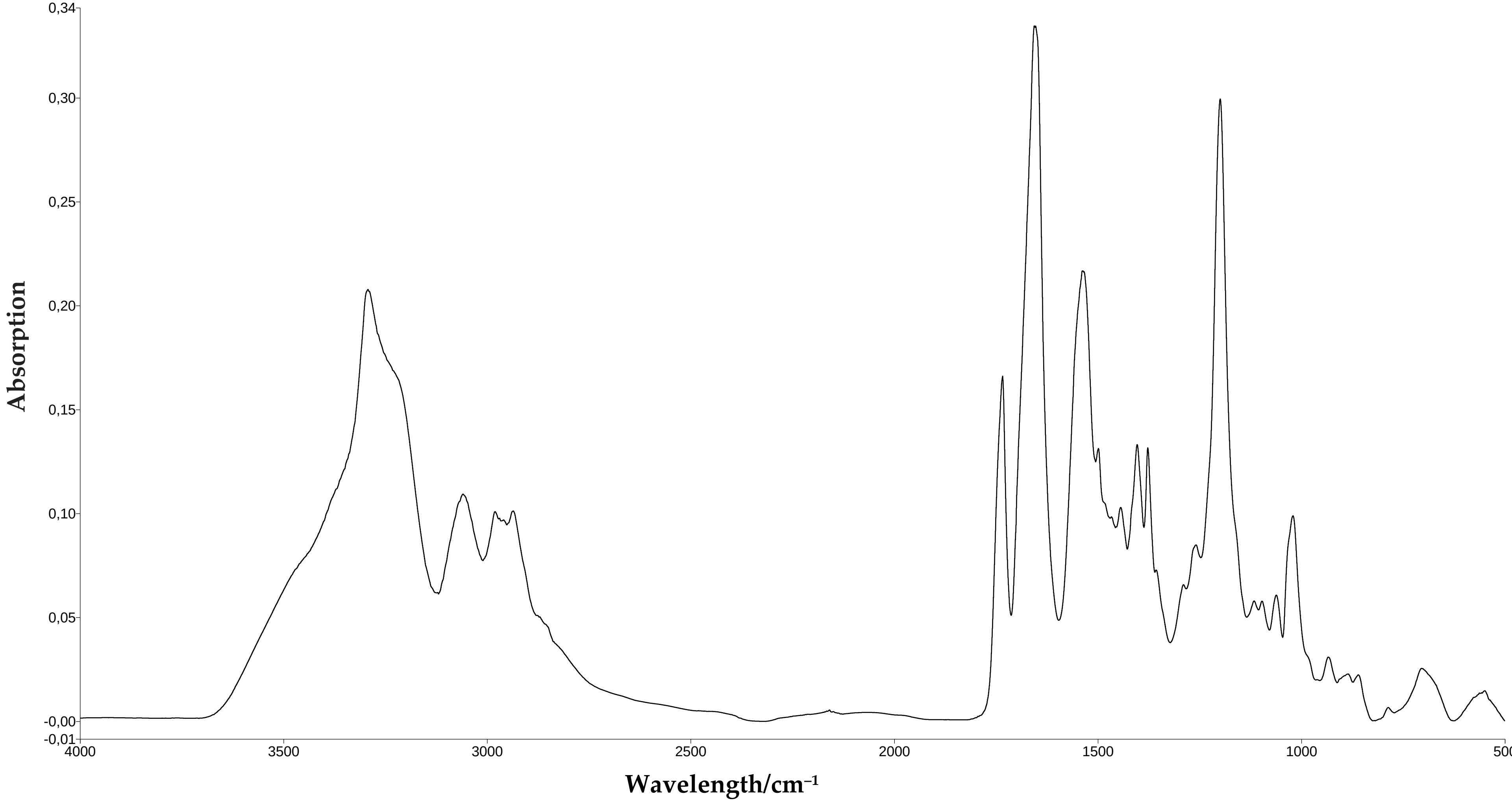

Fig. S29. IR spectrum of 4,8,14,18,23,26,28,31,32,35-deca-[(*N*-(3′,3′-dimethyl-3′-{(ethoxycarbonyl[*S*-benzyl]methyl)amidocarbonylmethyl}ammoniumpropyl)carbamoyl-methoxy]-pillar[5]arene decabromide *(15)*.

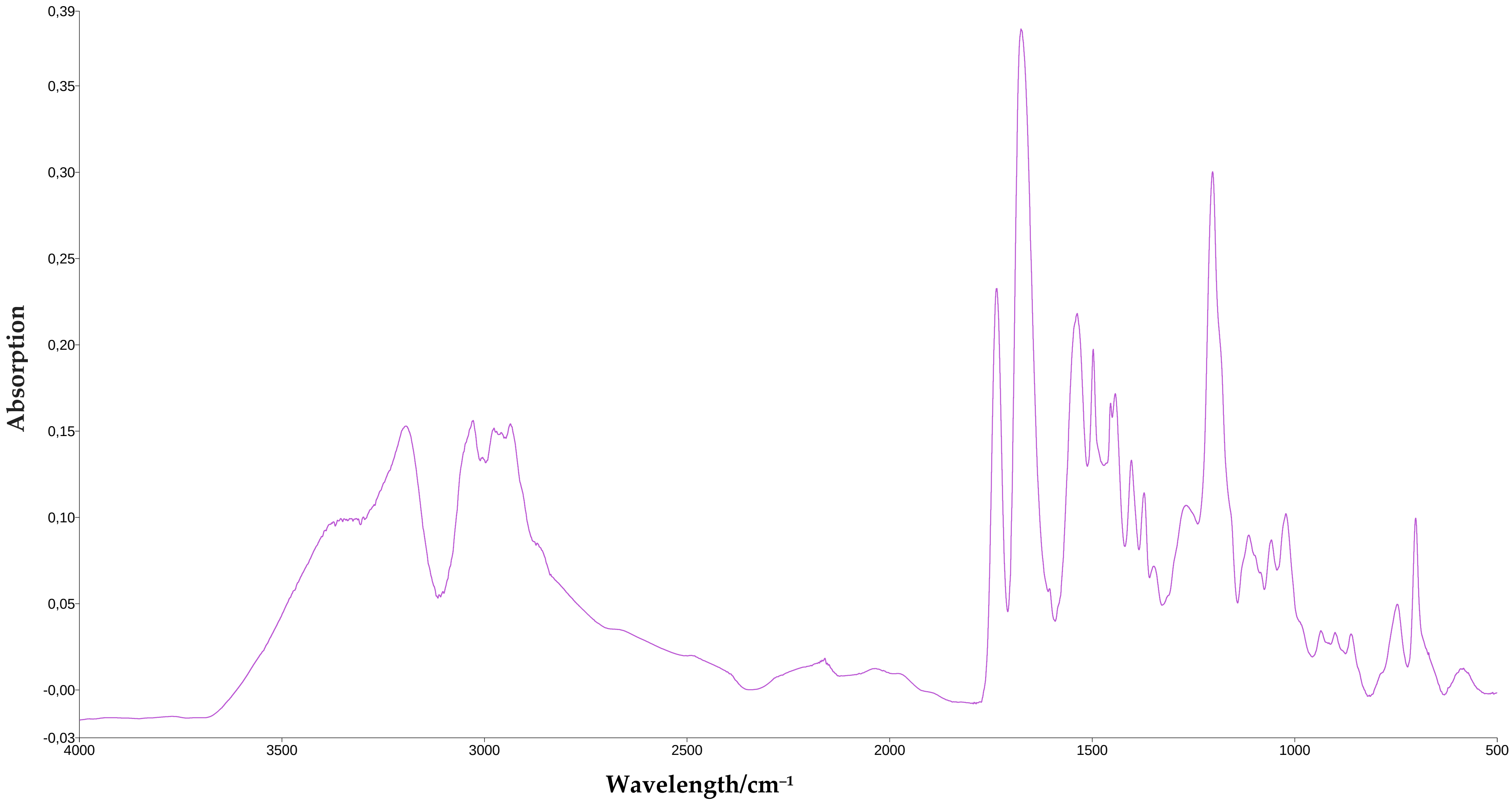

Fig. S30. IR spectrum of *N*-(2-((1-ethoxy-1-oxopropan-2-yl)amino)-2-oxoethyl)-3-(2-(4-methoxyphenoxy)acetamido)-*N*,*N*-dimethylpropan-1-ammonium bromide *(17)*.

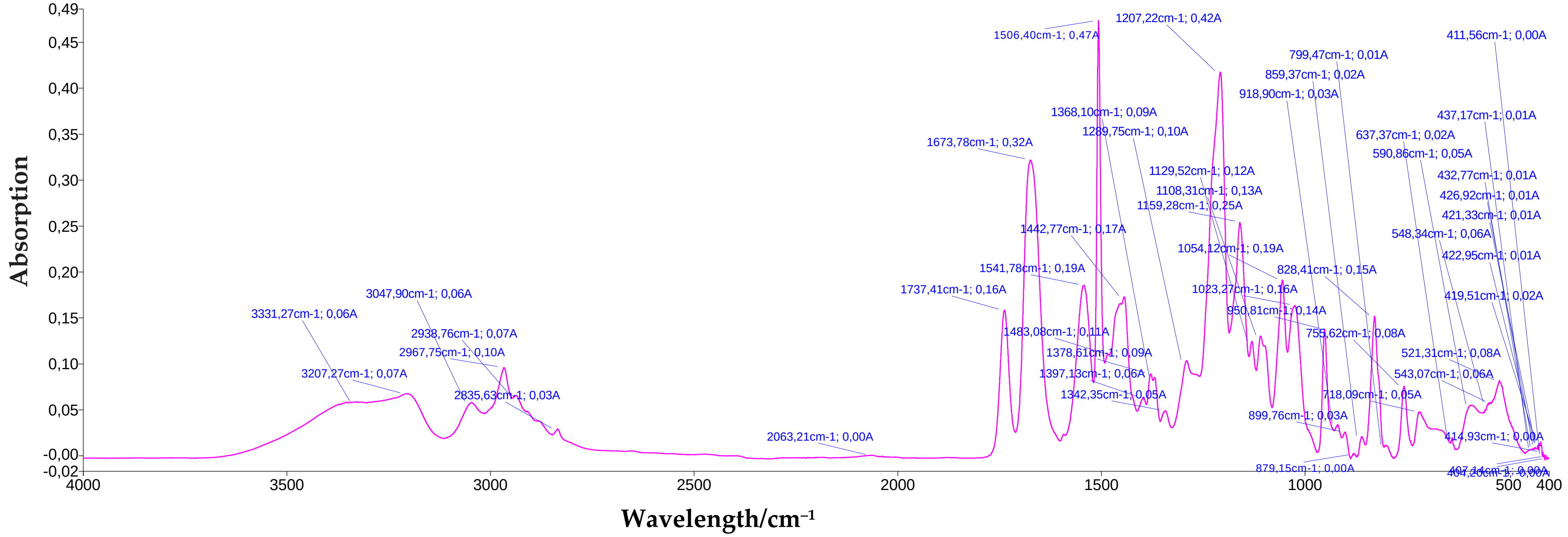

Fig. S31. IR spectrum of *N*-(2-((1-ethoxy-1-oxo-3-phenylpropan-2-yl)amino)-2-oxoethyl)-3-(2-(4-methoxyphenoxy)acetamido)-*N,N*-dimethylpropan-1-ammonium bromide *(18)*.

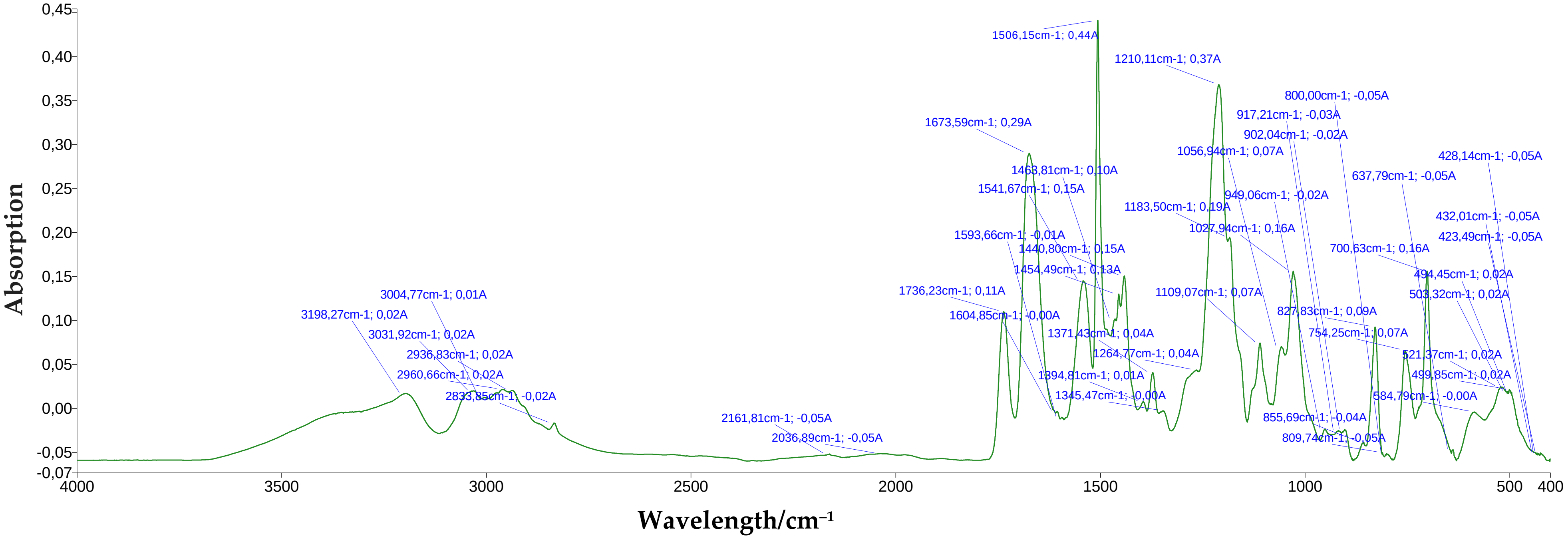

Fig. S32. 2D NMR NOESY ^{1}H-^{1}H spectrum of 4,8,14,18,23,26,28,31,32,35-deca-[(*N*-(3′,3′-dimethyl-3′-{(ethoxycarbonyl[*S*-methyl]methyl)amidocarbonylmethyl}ammoniumpropyl)carbamoyl-methoxy]-pillar[5]arene decabromide *(13)*, DMSO-d_6, 298 K, 400 MHz.

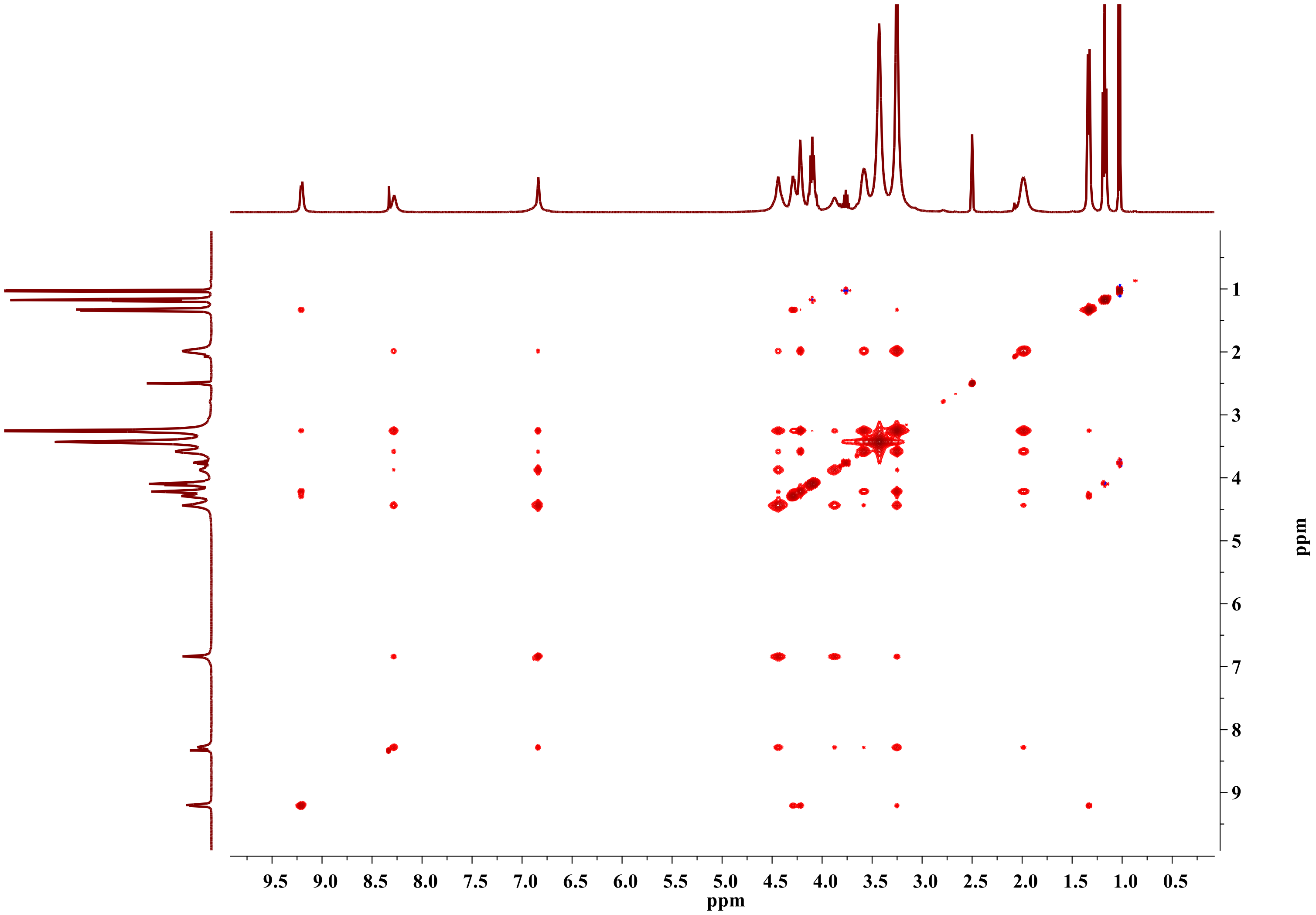

Fig. S33. 2D NMR NOESY ¹H-¹H spectrum of 4,8,14,18,23,26,28,31,32,35-deca-[(*N*-(3′,3′-dimethyl-3′-{(ethoxycarbonyl [*S*-benzyl]methyl)amidocarbonylmethyl}ammoniumpropyl)carbamoyl-methoxy]-pillar[5]arene decabromide *(15)*, DMSO-*d*₆, 298 K, 400 MHz.

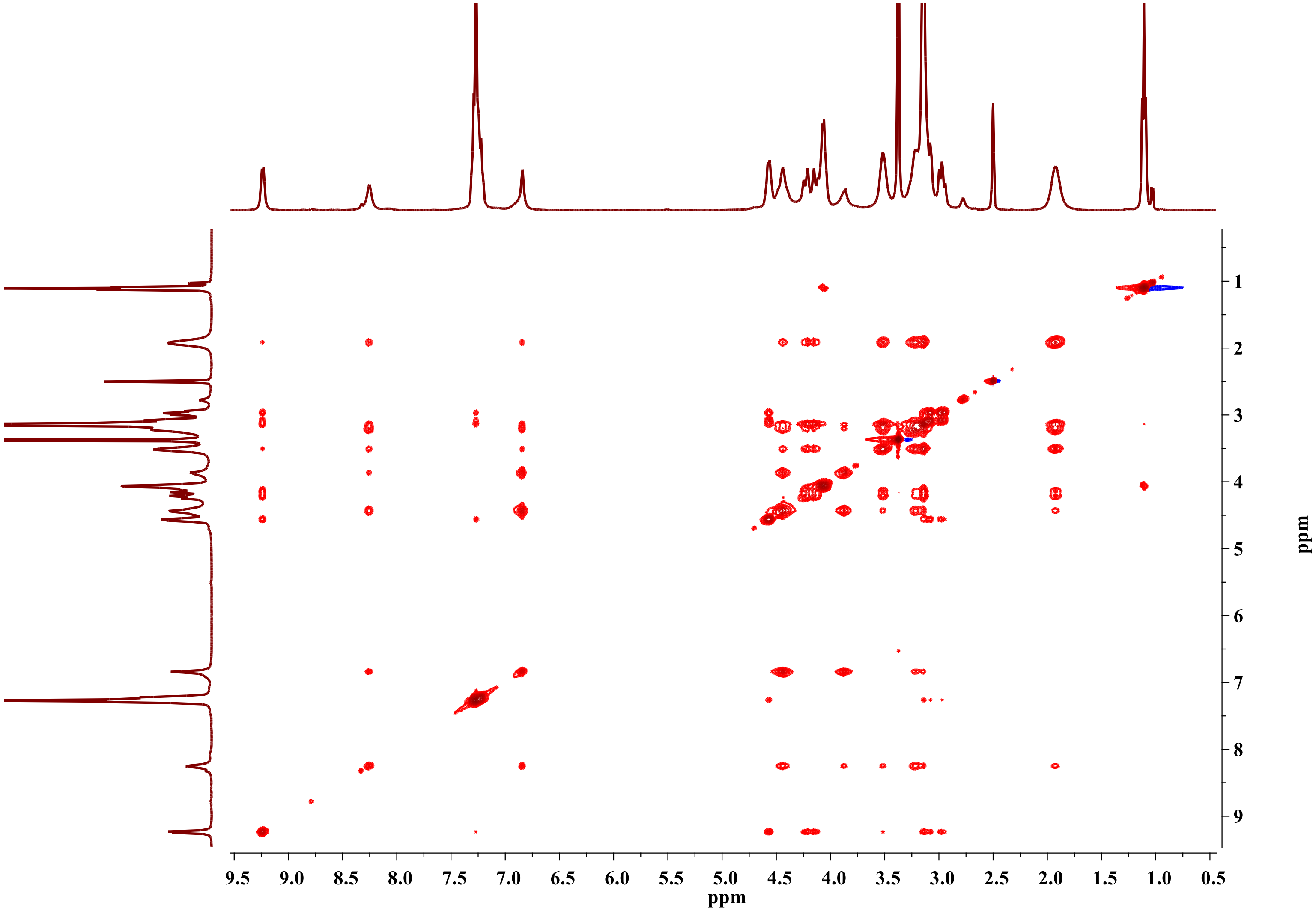

Fig. S34. CD spectra: a) 15 in DMSO (C = 1×10^{-4} M); b) 15 in water (C = 1×10^{-4} M); c) 13 in DMSO (C = 1×10^{-4} M); d) 13 in water (C = 1×10^{-4} M).

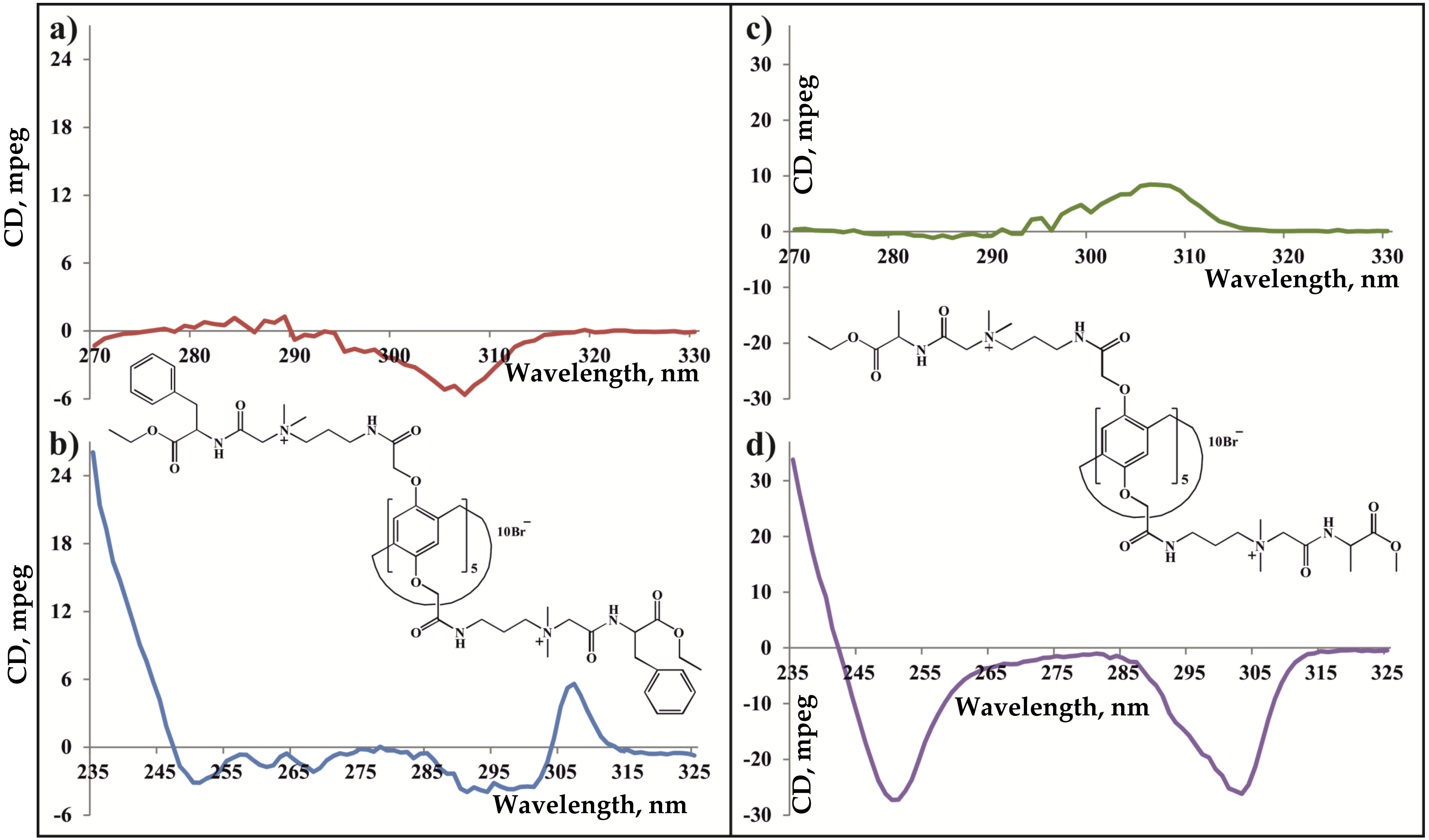

Fig. S35. CD spectra: a) 17 in DMSO (C = 5×10⁻⁴ M); b) 17 in water (C = 5×10⁻⁴ M); c) 18 in DMSO (C = 5×10⁻⁴ M); d) 18 in water (C = 5×10⁻⁴ M).

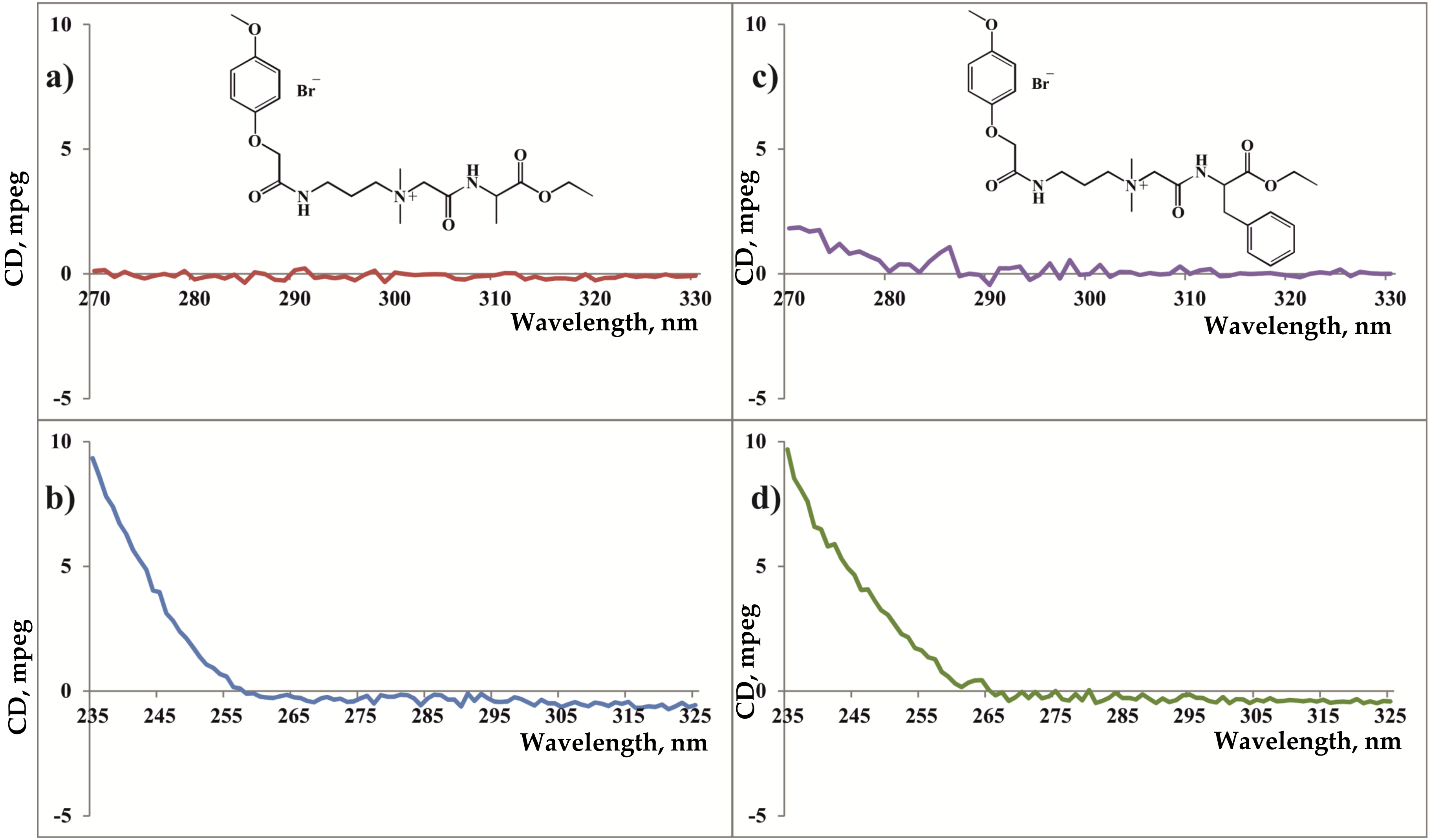

Determination of the stability constant and stoichiometry of the complex by the UV titration

The UV measurements were performed with "Shimadzu UV-3600" instrument. The $3 \cdot 10^{-3}$ M solution of the **G2** (30, 60, 90, 120, 150, 180, 210, 240, 270 and 300 µl) in DMSO was added to 0.3 ml of the solution of **13** ($3 \cdot 10^{-4}$ M) in DMSO and diluted to final volume of 3 ml with DMSO. The UV spectra of the solutions were then recorded. The stability constant of complexes were calculated by Bindfit. Three independent experiments were carried out for each series.

The UV measurements were performed with "Shimadzu UV-3600" instrument. The $3 \cdot 10^{-5}$ M solution of the **15** (300, 500, 600, 800, 900 1000, 1100, 1300, 1500 and 2000 µl) in water was added to 0.03 ml of the solution of **G4** ($1 \cdot 10^{-3}$ M) in water and diluted to final volume of 3 ml with water. The UV spectra of the solutions were then recorded. The stability constant of complexes were calculated by Bindfit. Three independent experiments were carried out for each series.

Fig. S36. a) UV-Vis spectra of mixtures of methyl orange G4 (1×10^{-5} M) with different concentrations of pillar[5]arene 15 in water; b) UV-Vis spectra of mixtures of pillar[5]arene 13 (3×10^{-4} M) with different concentrations of *S*-camphorsulfonic acid G2 in water.

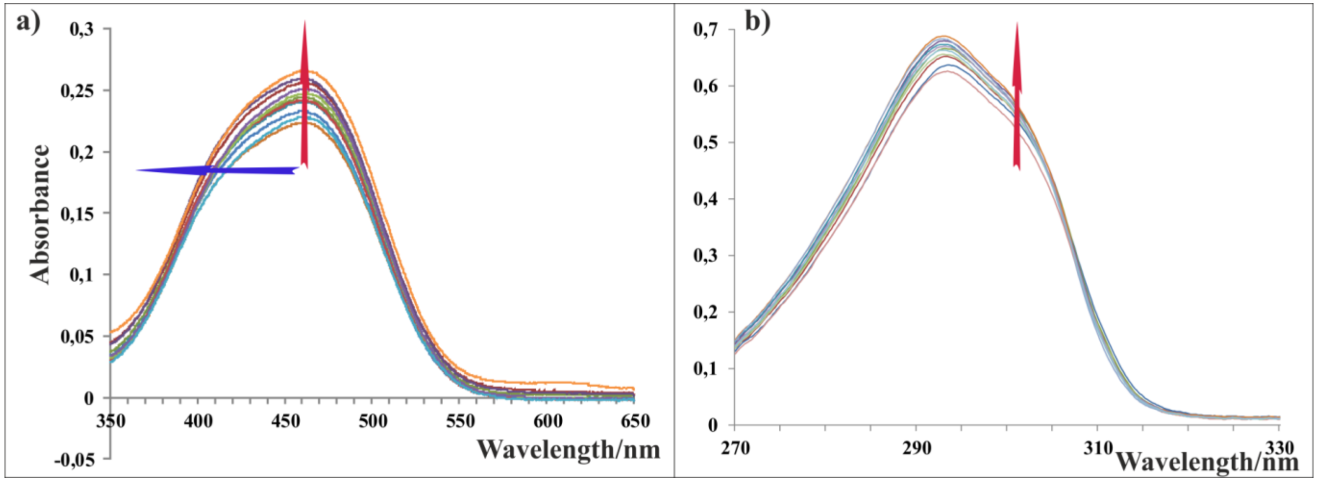

Fig. S37. Bindfit (Fit data to 1:1, 1:2 and 2:1 Host-Guest equilibria) Screenshots taken from the summary window of the website supramolecular.org. This screenshots shows the raw data for UV-vis titration of 13 with camphorsulfonic acid in DMSO, the data fitted to 1:1 binding model (a), 1:2 binding model (b) and 2:1 binding model (c).

(a)

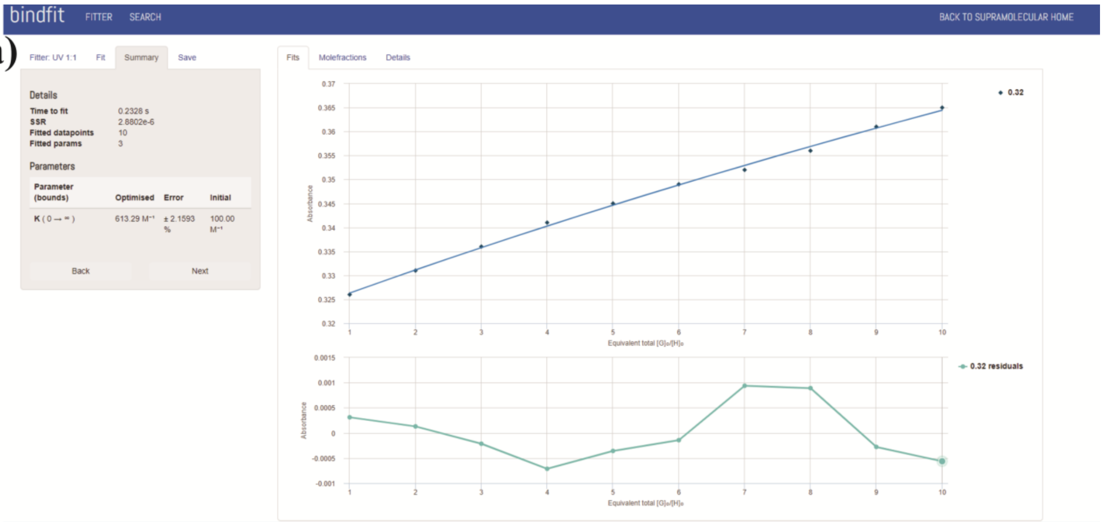

(b)

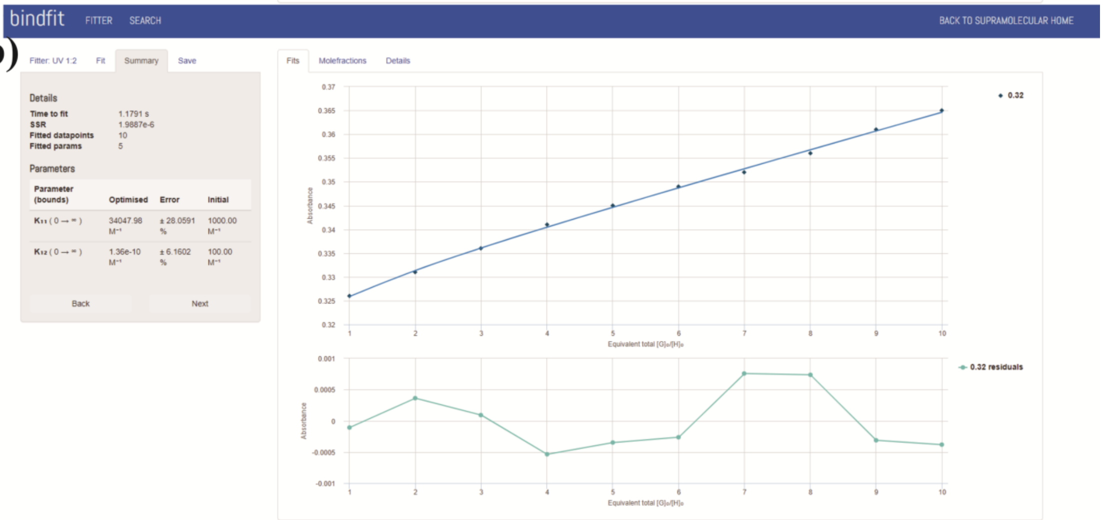

(c)

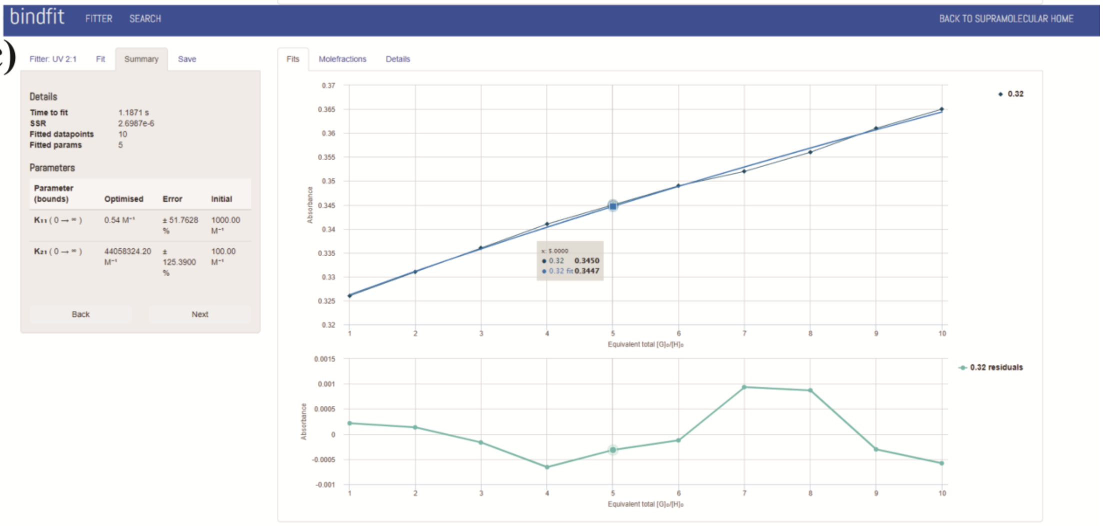

Fig. S38. Bindfit (Fit data to 1:1, 1:2 and 2:1 Host-Guest equilibria) Screenshots taken from the summary window of the website supramolecular.org. This screenshots shows the raw data for UV-vis titration of 15 with methyl orange in water, the data fitted to 1:1 binding model (a), 1:2 binding model (b) and 2:1 binding model (c).

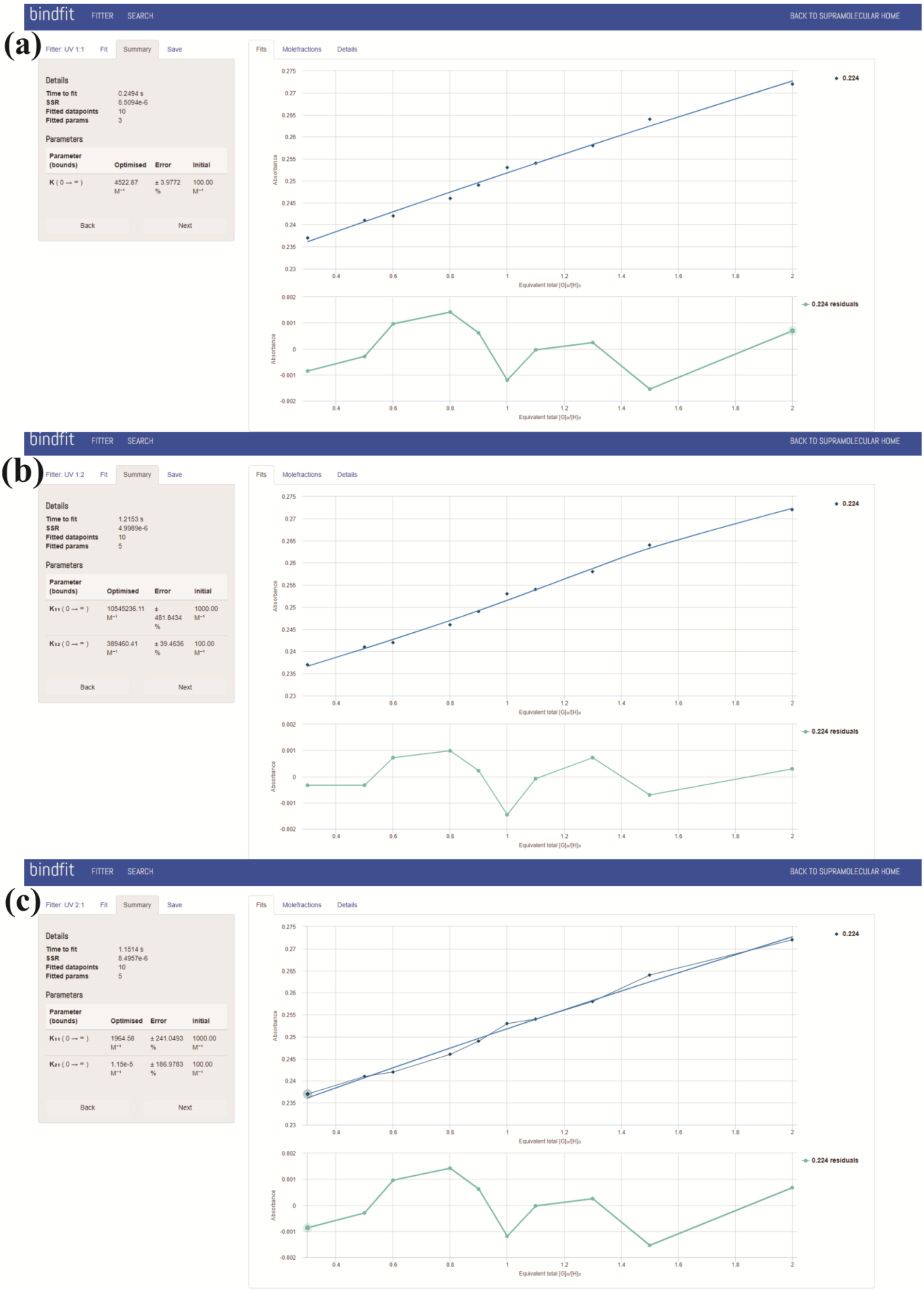

Job Plots

Series of the solutions of pillar[5]arene derivatives **13** and **15** and guests (camphorsulfonic acid and methyl orange) were prepared in DMSO (water). The volume ratio of the host and guest solutions varied from 0.6:2.4 to 2.4:0.6, respectively, with the total concentration of the host (H) and guest (G) being constant and equal to $3{\cdot}10^{-5}$ M. The solutions were used without further stirring. The absorbance A_i of the complexation systems was measured at the maximum absorbance wavelength of the complex. The absorbance values were used to plot a diagram from which maximum the structures of the complexes were deduced. Three independent experiments were carried out for each system.

Fig. S39. a) the Job's plot for the determination of the stoichiometry in the complex of the system pillar[5]arene 13 and camphorsulfonic acid in DMSO;

b) the Job's plot for the determination of the stoichiometry in the complex of the system pillar[5]arene 15 and methyl orange in water.

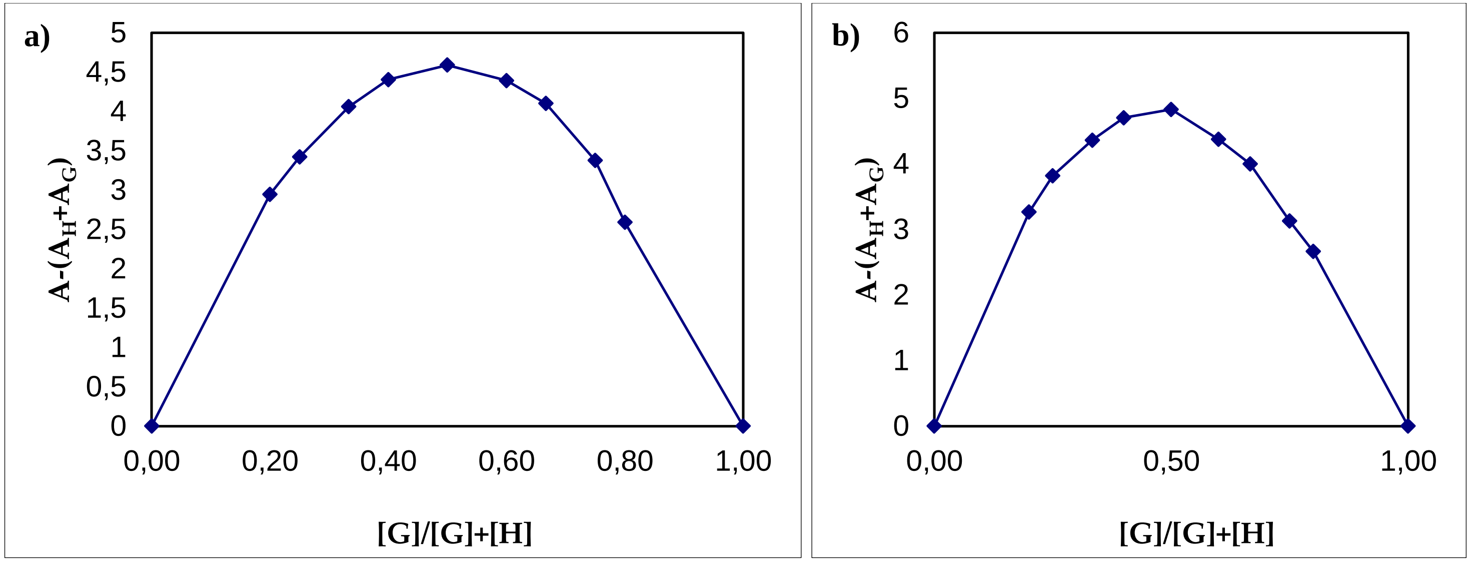

Fig. S40. Fragment of ^{1}H NMR spectra (DMSO-d_6, 298 K, 400 MHz): a) G2 (1×10^{-2} M); b) 13 (1×10^{-2} M) + G2 (1×10^{-2} M); c) 13 (1×10^{-2} M).

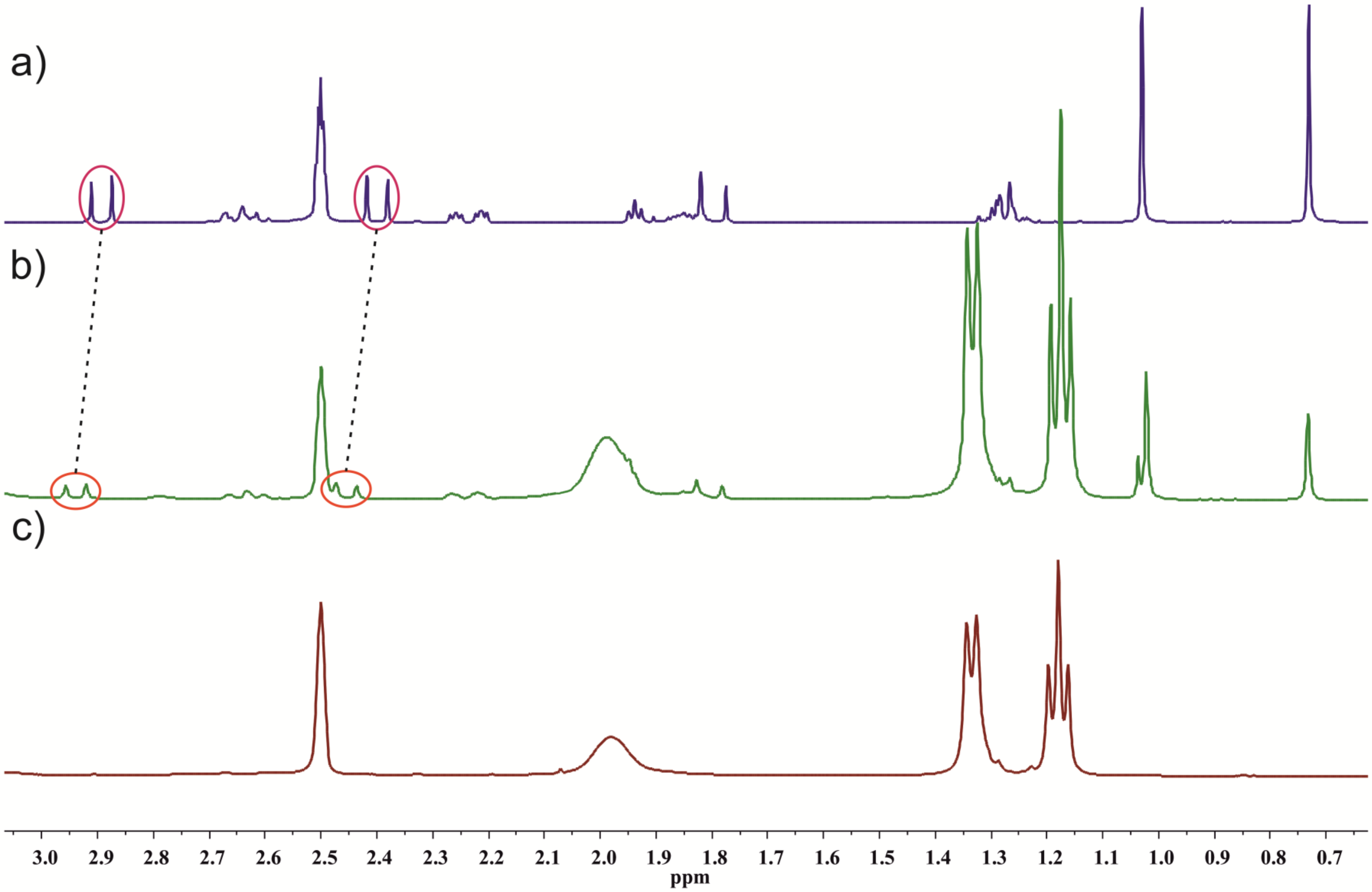

Fig. S41. ^{1}H **NMR (DOSY) spectrum of 13 (1×10^{-2} M) and camphorsulfonic acid (1×10^{-2} M), DMSO-d_6, 298 K, 400 MHz.**

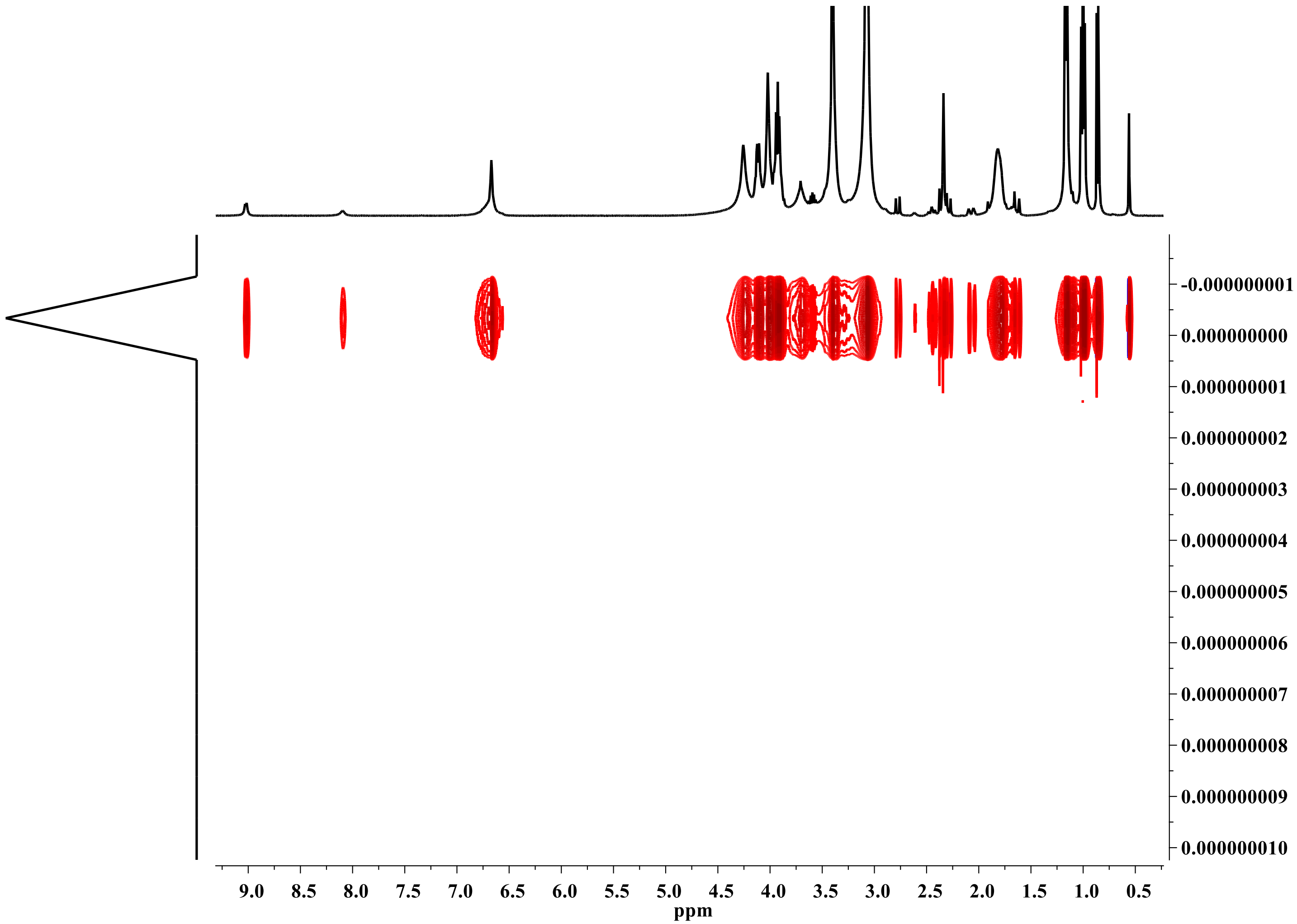

Table S1. Diffusion coefficients of pillar[5]arene 13, *S*-camphorsulfonic acid G2 and 13/G2 complex in DMSO-d_6 (400 MHz, 298 K).

Compounds	D (10^{-10} m^2s^{-1})
**13**	3.96
**G2**	2.07
**13/G2**	0.45

Fig. S42. Size distribution of self-assembles of macrocycle 12 at C = 1×10^{-4} M in water.

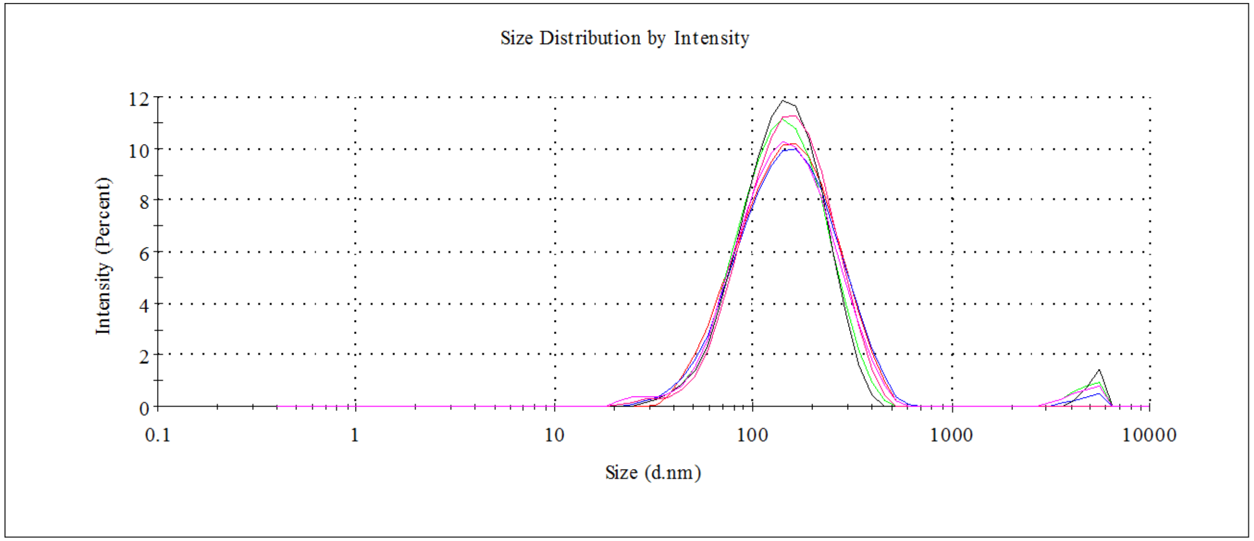

Fig. S43. Size distribution of self-assembles of macrocycle 13 at C = 1×10^{-3} M in water.

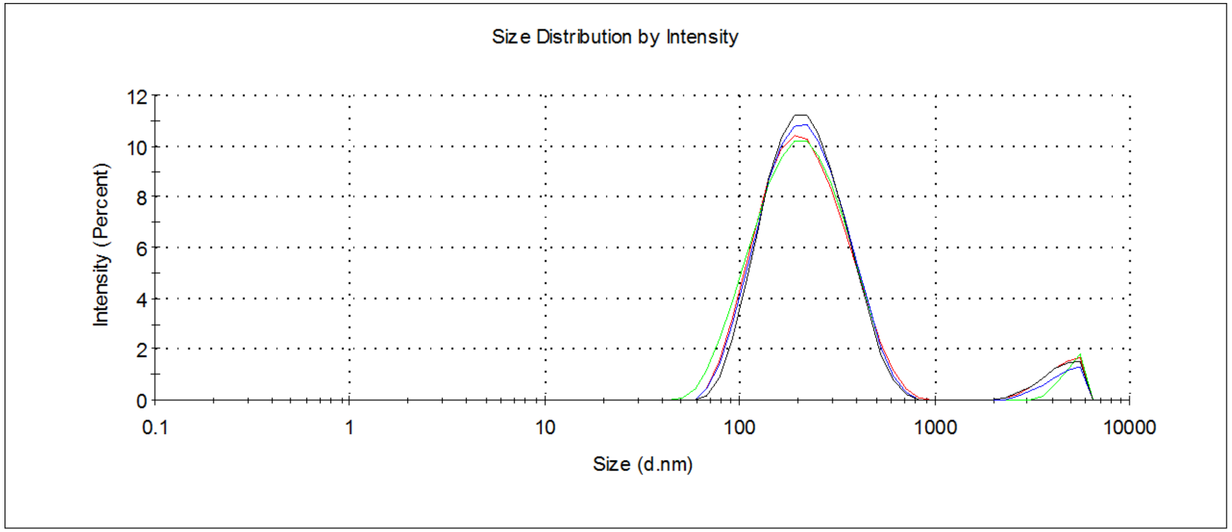

Fig. S44. Size distribution of self-assembles of macrocycle 13 and camphorsulfonic acid at C = 1×10^{-5} M in DMSO.

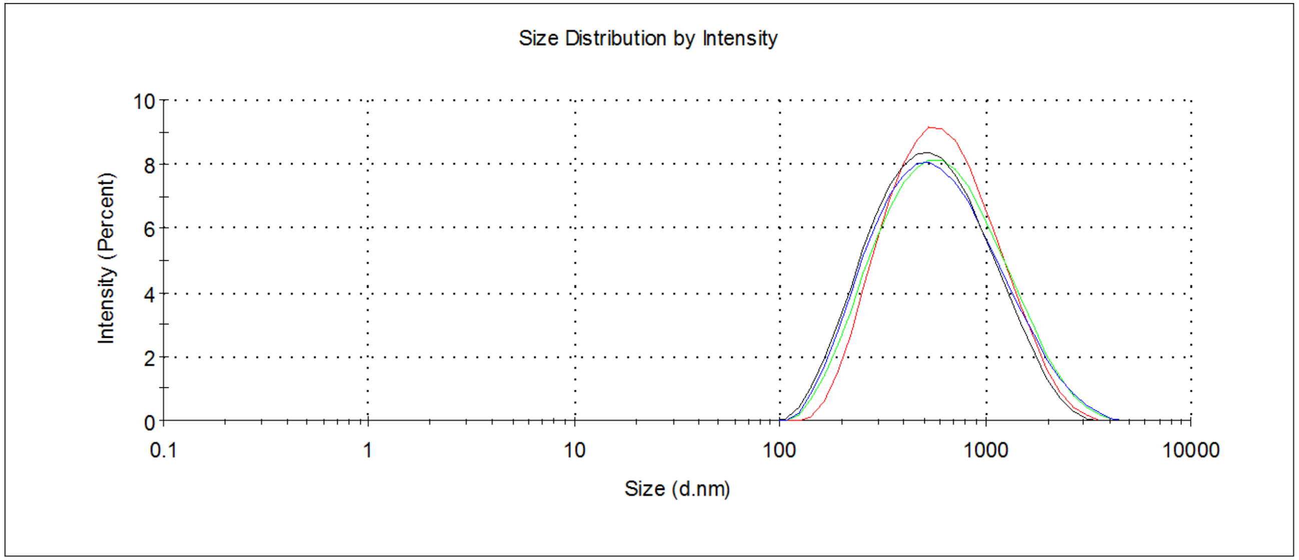

Fig. S45. CD spectra of 13, 1×10^{-4} (green), G2, 1×10^{-3} (purple) and 13, 1×10^{-4} + G2, 1×10^{-3} (red) in water.

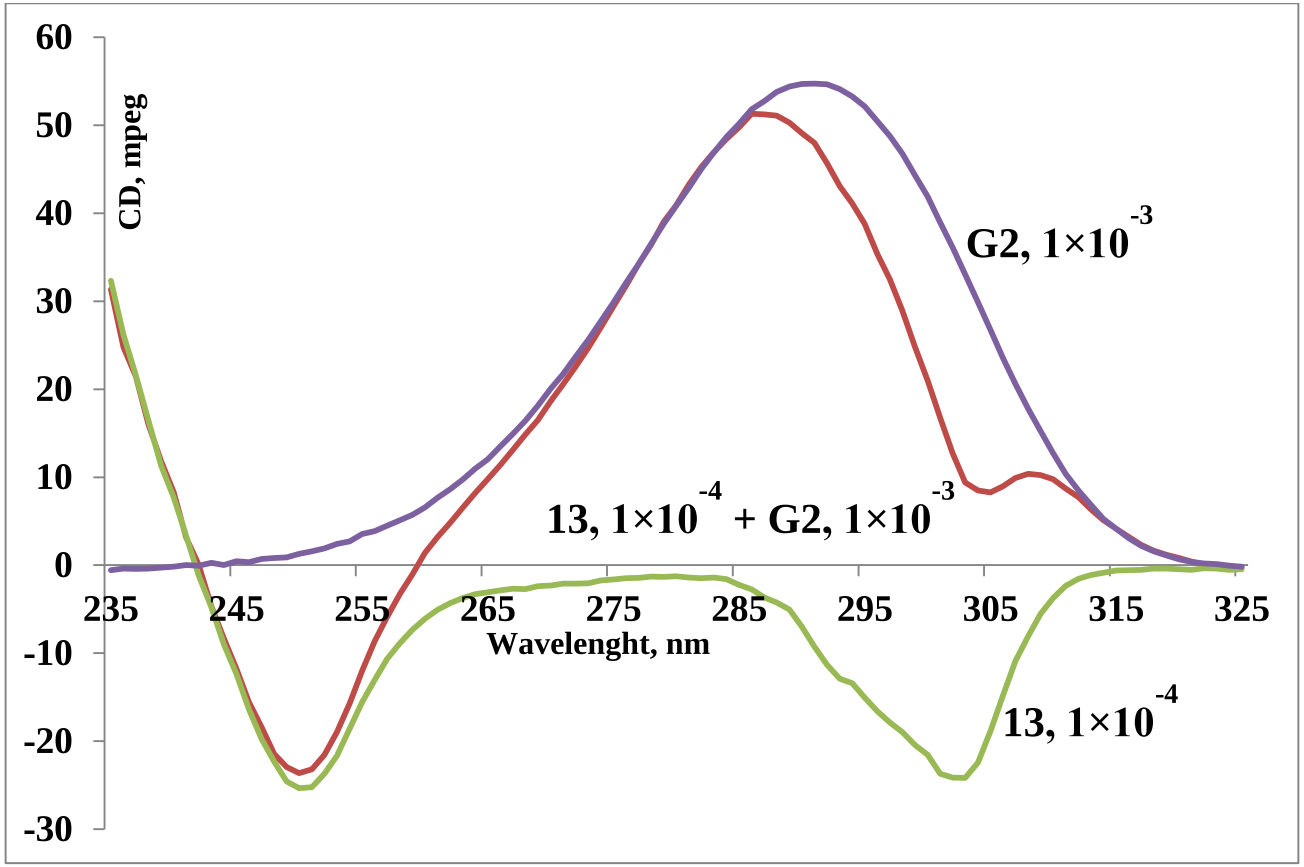

Fig. S46. CD spectra of 13, 1×10^{-4} (green), G2, 1×10^{-3} (purple) and 13, 1×10^{-4} + G2, 1×10^{-3} (red) in DMSO.

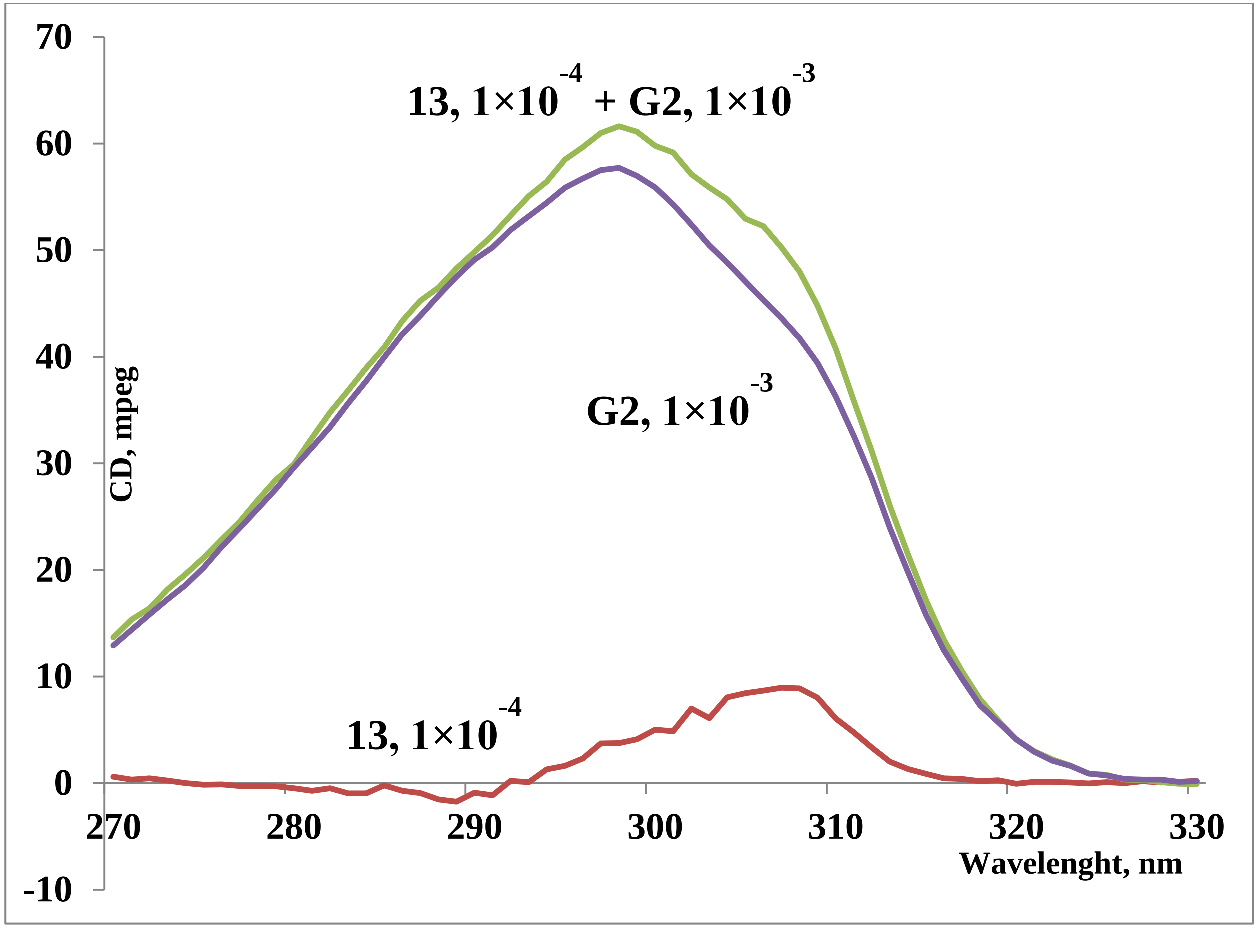

Fig. S47. TEM images (water, C = 1×10^{-4} M) of: (A) compound 12; (B) compound 13.

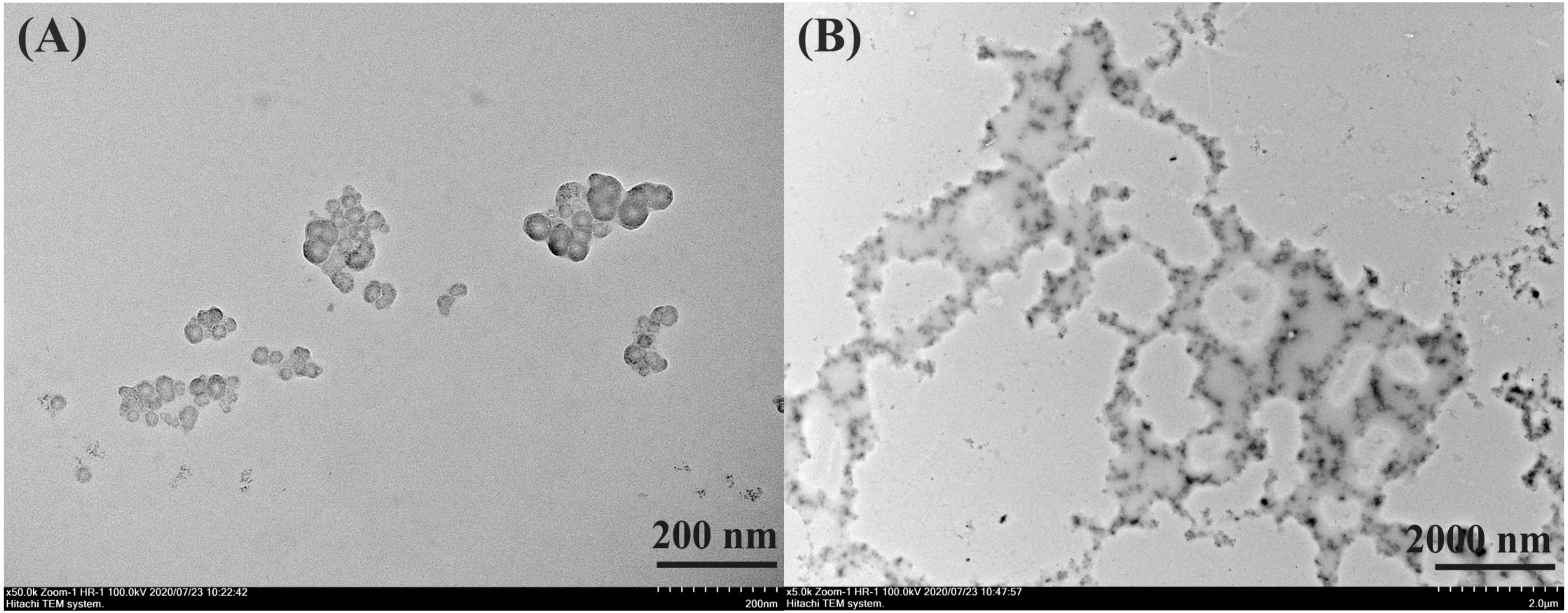

Supplement: Supplementary file 1 [file ijms-21-07206-s001.pdf]
